# New miRNA Signature Heralds Human NK Cell Subsets at Different Maturation Steps: Involvement of miR-146a-5p in the Regulation of KIR Expression

**DOI:** 10.3389/fimmu.2018.02360

**Published:** 2018-10-15

**Authors:** Silvia Pesce, Margherita Squillario, Marco Greppi, Fabrizio Loiacono, Lorenzo Moretta, Alessandro Moretta, Simona Sivori, Patrizio Castagnola, Annalisa Barla, Simona Candiani, Emanuela Marcenaro

**Affiliations:** ^1^Department of Experimental Medicine (DIMES), University of Genoa, Genoa, Italy; ^2^Department of Informatic Bioengeneering, Robotic and System Engeneering, University of Genoa, Genoa, Italy; ^3^Centre of Excellence for Biomedical Research (CEBR), University of Genoa, Genoa, Italy; ^4^Immunology Operative Unit, IRCCS San Martino Polyclinical Hospital, Genoa, Italy; ^5^Department of Immunology, IRCCS Bambino Gesù Children's Hospital, Rome, Italy; ^6^Department of Integrated Oncological Therapies, IRCCS San Martino Polyclinical Hospital, Genoa, Italy; ^7^Department of Earth Science, Environment and Life (DISTAV), University of Genoa, Genoa, Italy

**Keywords:** NK cells, miRNA, KIR, CD56^bright^, CD56^dim^, NK cell subsets, miRNA microarrays, multivariate statistical analysis

## Abstract

Natural killer cells are cytotoxic innate lymphoid cells that play an important role for early host defenses against infectious pathogens and surveillance against tumor. In humans, NK cells may be divided in various subsets on the basis of the relative CD56 expression and of the low-affinity FcγRIIIA CD16. In particular, the two main NK cell subsets are represented by the CD56^bright^/CD16^−/dim^ and the CD56^dim^/CD16^bright^ NK cells. Experimental evidences indicate that CD56^bright^ and CD56^dim^ NK cells represent different maturative stages of the NK cell developmental pathway. We identified multiple miRNAs differentially expressed in CD56^bright^/CD16^−^ and CD56^dim^/CD16^bright^ NK cells using both univariate and multivariate analyses. Among these, we found a few miRNAs with a consistent differential expression in the two NK cell subsets, and with an intermediate expression in the CD56^bright^/CD16^dim^ NK cell subset, representing a transitional step of maturation of NK cells. These analyses allowed us to establish the existence of a miRNA signature able to efficiently discriminate the two main NK cell subsets regardless of their surface phenotype. In addition, by analyzing the putative targets of representative miRNAs we show that hsa-miR-146a-5p, may be involved in the regulation of killer Ig-like receptor (KIR) expression. These results contribute to a better understanding of the physiologic significance of miRNAs in the regulation of the development/function of human NK cells. Moreover, our results suggest that hsa-miR-146a-5p targeting, resulting in KIR down-regulation, may be exploited to generate/increment the effect of NK KIR-mismatching against HLA-class I^+^ tumor cells and thus improve the NK-mediated anti-tumor activity.

## Introduction

Natural killer (NK) cells are crucial effectors in the immune response against pathogen infection, in tumor surveillance and in regulating immune homeostasis (1). NK cells develop in the bone marrow and complete their maturation in peripheral organs. The total population of human NK cells is phenotypically and functionally heterogeneous. In physiological conditions, the distribution/intensity of the surface markers CD56 and CD16 (FcγRIIIA) defines two NK lymphocytes subsets: the CD56^bright^/CD16^−/dim^ population that makes up about 10% of peripheral blood NK cells and the CD56^dim^/CD16^bright^ subset that represents about 90% of circulating NK cells (2). The CD56^bright^/CD16^−^ NK cell subset represents 50–70% of CD56^bright^, whereas the CD56^bright^/CD16^dim^ cells are ~30–50% of CD56^bright^ (3, 4). Additional NK cell populations exist but are a minority in healthy individuals. These include the CD56^dim^/CD16^−^ (whose function is largely unknown) (5) and the functional CD56^−^/CD16^bright^ NK cell subsets. This last subset is often expanded during certain viral infections, but it is usually hyporesponsive under these conditions (6–9).

CD56^bright^ NK cells exert limited cytotoxic capacity, but produce abundant cytokines such as IFN-γ, TNF-α, and GM-CSF. For this ability, this subset has been generally referred to as regulatory NK cells. Conversely, CD56^dim^ NK cells are highly cytotoxic (they contain much more perforin, granzymes in cytolytic granules) and play a key role in natural and Ab-mediated cell cytotoxicity. Nevertheless, subsequent data indicated that they can also produce relatively high amounts of pro-inflammatory cytokines following the engagement of activating NK receptors (10). The different functional outcomes of CD56^bright^ and CD56^dim^ NK cells are dependent on different repertoires of activating (11) and inhibitory receptors (12) and on distinct homing capacities associated with different expression of chemokine receptors on their surface. Indeed, CD56^bright^ and CD56^dim^ NK cell subsets differ in their pattern of expression of inhibitory NK cell receptors specific for HLA-class I molecules (13). In humans, different types of inhibitory receptors specific for HLA-class I molecules exist: (i) killer Ig-like receptors (KIRs/CD158), that recognize the polymorphic HLA-A, -B, and -C molecules (12, 14, 15), (ii) the immunoglobulin-like receptor 1 (LIR-1/ILT-2/CD85j), that is specific for different HLA-class I molecules (16), and (iii) the CD94/NKG2A (CD94/CD159a) heterodimer, that recognizes HLA-E (17), a non-polymorphic non-classical HLA molecule.

CD56^bright^ NK cells are KIR^−^ and CD94/NKG2A^+^, whereas CD56^dim^ NK cells are KIR^+^ and/or CD94/NKG2A^+^ (12, 14). Moreover, CD56^dim^ NK cells express CXCR1, CX3CR1, and ChemR23 receptors, and therefore mainly migrate to inflamed peripheral tissues, while the CD56^bright^ subset expresses CCR7 and homes primarily to secondary lymphoid organs (18). Accordingly, CD56^bright^ NK cells are 10 times more frequent in normal lymph nodes than in peripheral blood (19, 20). Remarkably, recent data indicate that in pathological conditions that include viral infections or tumors the CD56^dim^ subset may also *de novo* express CCR7 and migrate toward lymph nodes (21–25).

A current hypothesis regarding their development indicates that immature CD56^bright^ NK cells (that are consistently CD94/NKG2A^+^), are precursors of CD56^dim^ (8). During their differentiation process, NK cells up-regulate CD16 developing from CD56^bright^/CD16^−^ into CD56^bright^/CD16^dim^ and then into CD56^dim^/CD16^bright^ NK cells. In turn, CD56^dim^ NK cells change their phenotypic features losing expression of CD94/NKG2A, and successively acquiring the KIRs and LIR-1 inhibitory receptors. The terminally differentiated phenotype of CD56^dim^ cells is characterized by CD57 expression that is associated with poor reactivity to cytokine stimulation, but maintains cytolytic capacity (26, 27). Such linear differentiation is supported by the evidence that (i) CD56^bright^ NK cells have longer telomeres than CD56^dim^ NK cells, (ii) they are more represented in peripheral blood early after hematopoietic stem cell (HSC) transplantation, and (iii) they differentiate into CD56^dim^ NK cells in humanized mice engrafted with human HSC (28, 29). However, despite the known phenotypic and functional differences between the two main NK cell subsets, many cellular and molecular features governing the transition from CD56^bright^ to CD56^dim^ cells remain unknown.

microRNAs (miRNAs) are a large family of small non-coding RNAs that target the 3′-UTR of mRNAs, thereby reducing mRNA stability and/or limiting protein translation, consequently regulating critical cellular processes (30, 31). Recent studies have identified miRNAs expressed in resting and activated mouse and human NK cells, and have shown that global miRNA deficiency results in altered mature NK cell functional responses *in vitro* and *in vivo*. In particular, it has been demonstrated that miRNAs regulate fundamental NK cell processes including activation in terms of cytokine production, cytotoxicity, and proliferation, but probably also their development and maturation (32–35). Thus, we decided to analyze miRNA expression in different NK cell subsets and perform a functional annotation and prediction of putative mRNA targets in order to highlight any differences that might be correlated with their subsequent maturation steps.

Our analysis allowed us to establish the existence of a miRNA signature able to discriminate the two main NK cell subsets, regardless of their surface phenotype. Moreover, we also identified miRNAs exclusively expressed by CD56^bright^/CD16^−^ NK cells (hsa-miR-31a-5p and hsa-miR-130a-5p), or by CD56^dim^ NK cells (hsa-miR-181a-2-3p). Notably, these miRNAs show an intermediate expression in CD56^bright^/CD16^dim^ NK cells. These data strengthen the hypothesis that CD56^bright^/CD16^−^ NK cells mature toward CD56^dim^ NK cells through an intermediate step represented by CD56^bright^/CD16^dim^ NK cells.

Importantly, our results also revealed that hsa-miR-146a-5p, over-expressed in CD56^bright^ NK cells, is involved in the modulation of KIR expression, and this could result in possible implications in the control of NK cell licensing and cytotoxicity (36).

Here we show for the first time differentially expressed miRNAs in human NK cell subsets, thus providing valuable clues for further explanation of miRNA regulation in human NK cell maturation and suggesting their potential exploitation in NK cell immunotherapy.

## Results

### Univariate statistical method for miRNAs signature identification

**Figure 1:**
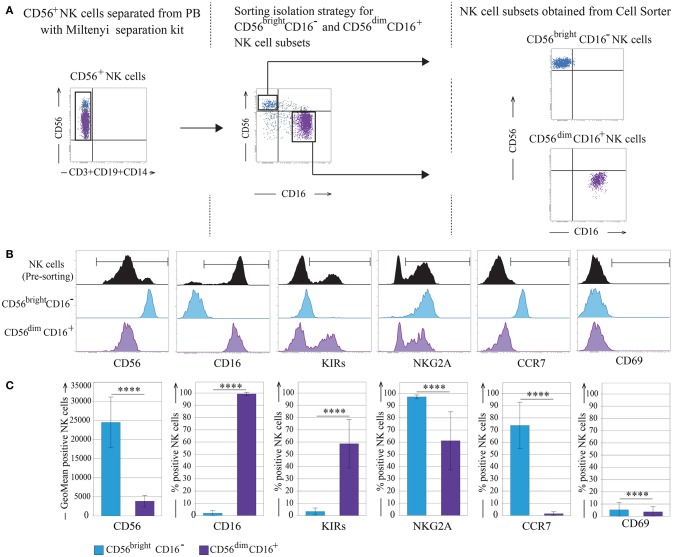
CD56^bright^ and CD56^dim^ NK cell subsets isolation strategy. **(A)** Sorting separation strategy for CD56^bright^/CD16^−^ and CD56^dim^/CD16^+^ NK cells subsets. **(B)** Informative NK cell surface markers pre- and post-sorting are shown (one representative donor). **(C)** Post-sorting statistical analysis of informative NK cells surface markers. Data represents the mean ± SD, *n* = 10 donors. *T*-test analysis comparing CD56^bright^/CD16^−^ and CD56^dim^/CD16^+^ NK cell subsets indicated as ****p < 0.0001. Color legend: Pre-sorting NK cells are indicated in black, CD56^bright^/CD16^−^ NK cells are indicated in light blue and CD56^dim^/CD16^+^ NK cells are indicated in purple.

NK cells were negatively isolated from fresh peripheral blood mononuclear cells (PBMC) derived from 10 different healthy donors and then the most immature CD56^bright^/CD16^−^ (referred as CD56^bright^) and the mature CD56^dim^/CD16^bright^ (referred as CD56^dim^) NK cell subsets were sorted by the expression of CD56 and CD16 and analyzed by flow cytometry for different markers in order to control their purity and phenotypic features (Figures 1A–C). As expected, CD56^bright^ NK cells were homogeneously CD16^−^, KIR^−^, NKG2A^+^, and CCR7^+^, whereas CD56^dim^ NK cells were CD16^+^, KIR^+^ and/or NKG2A^+^, and CCR7^−^. This analysis confirmed the high purity of the two sorted cell subpopulations. Importantly, in all instances, both these NK cell subsets did not express the activation marker CD69 (Figures 1B,C).

Then, miRNA expression profiles of human CD56^bright^ and CD56^dim^ NK cells subsets were investigated with the human miRNA microarray kit v19.0, which allows for the detection of a total of 2006 different human miRNA sequences (miRBase v19.0). In particular, 10 separate samples for each of CD56^bright^ and CD56^dim^ (20 total samples) were hybridized on the arrays. The entire dataset was composed of two separately generated microarray datasets that were analyzed together (Supplementary File 1).

The normalized batch-corrected log_2_ intensity values were used to identify miRNAs differentially expressed between the CD56^bright^ and the CD56^dim^ NK cell populations (see also Materials and Methods). Considering an arbitrary threshold of 1 light unit (1LU), 251 miRNAs were over the threshold in half or more of the 10 CD56^bright^ samples, 198 of which were over the threshold in all 10 CD56^bright^ samples. Similarly, 262 miRNAs were over the threshold in half or more of the 10 CD56^dim^ samples, 213 of which were over the threshold in all 10 CD56^dim^ samples. Interestingly, 232 miRNAs were detectable in both NK cell subsets and only 49 were expressed in only CD56^bright^ (19 miRNAs) or CD56^dim^ (30 miRNAs).

**Figure 2:**
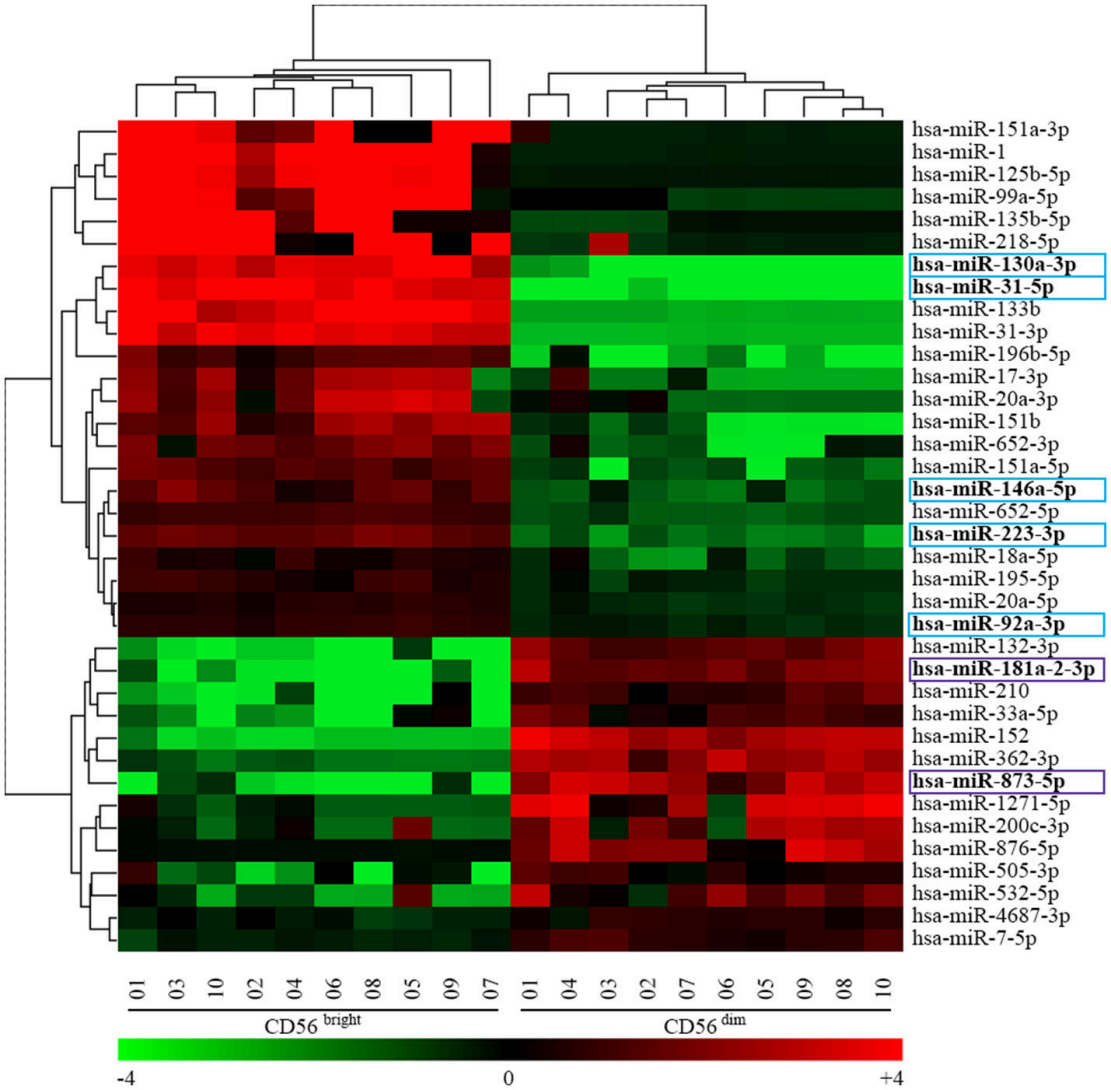
Heatmap representing the unsupervised hierarchical clustering analysis of CD56^bright^/CD16^−^ and CD56^dim/^CD16^+^ NK cells miRNAs signature obtained with the univariate analysis. Heatmap of 37 miRNAs signature identified by the human miRNA microarray kit v19.0. Dendrograms indicate the results of the bi-clustering analysis. Table at the bottom of the figure helps with the translation between centered log_2_ data and fold-change values, and also introduces the heat map color scheme used. Colored boxes indicate 7 of the most regulated miRNAs. Color legend: the most up-regulated miRNAs on CD56^bright^/CD16^−^ NK cells are indicated in light blue and the most up-regulated miRNAs on CD56^dim^/CD16^+^ NK cells are indicated in purple.

Figure 2 shows the two-color heatmap plot as result of the unsupervised hierarchical clustering in which we performed a bi-clustering analysis of both miRNA and NK samples. This analysis clearly separates miRNAs differentially expressed and at the same time CD56^bright^ from CD56^dim^ NK cell subset. At the same time, we provided evidence that it is possible to separate the two main NK cell subsets by unsupervised hierarchical clustering. In particular, Figure 2 represents a two-color heatmap plot depicting the results of the bi-clustering analysis of both miRNA and NK samples. This analysis clearly separetes CD56^bright^ from CD56^dim^ NK cell subset. In particular, the heatmap identify a first level of signature characterized by 14 up-regulated and 23 down-regulated miRNAs in the CD56^dim^ NK cell population. Importantly, among the most regulated miRNAs, we found 5 miRNAs deeply and homogeneously down-regulated (hsa-miR-31-5p, hsa-miR-130a-3p, hsa-miR-223-3p, hsa-miR-146a-5p, and hsa-miR-92a-3p) and 2 miRNAs deeply and homogeneously up-regulated (hsa-miR-873-5p and hsa-miR-181a-2-3p) in all the CD56^dim^ samples compared to CD56^bright^ ones (see arrowheads in Figure 2). Notably hsa-miR-31-5p and hsa-miR-130a-3p expression was below detectable levels on CD56^dim^ NK cells, whereas hsa-miR-181a-2-3p expression was below detectable levels on CD56^bright^ NK cells. These miRNAs may be considered specific for CD56^bright^ or CD56^dim^ NK cells, respectively.

### A multivariate statistical method for miRNAs signature identification

To better capture the interplay among the miRNAs in the two cell groups (i.e., CD56^dim^ and CD56^bright^), in addition to the univariate analysis shown in Figure 2, we performed a multivariate analysis by using the l1l2-penalized regularization method, a machine learning technique for variable selection. A schematic drawing in Supplementary Figure 2A shows the approach used in such analysis (see also Materials and Methods).

**Figure 3:**
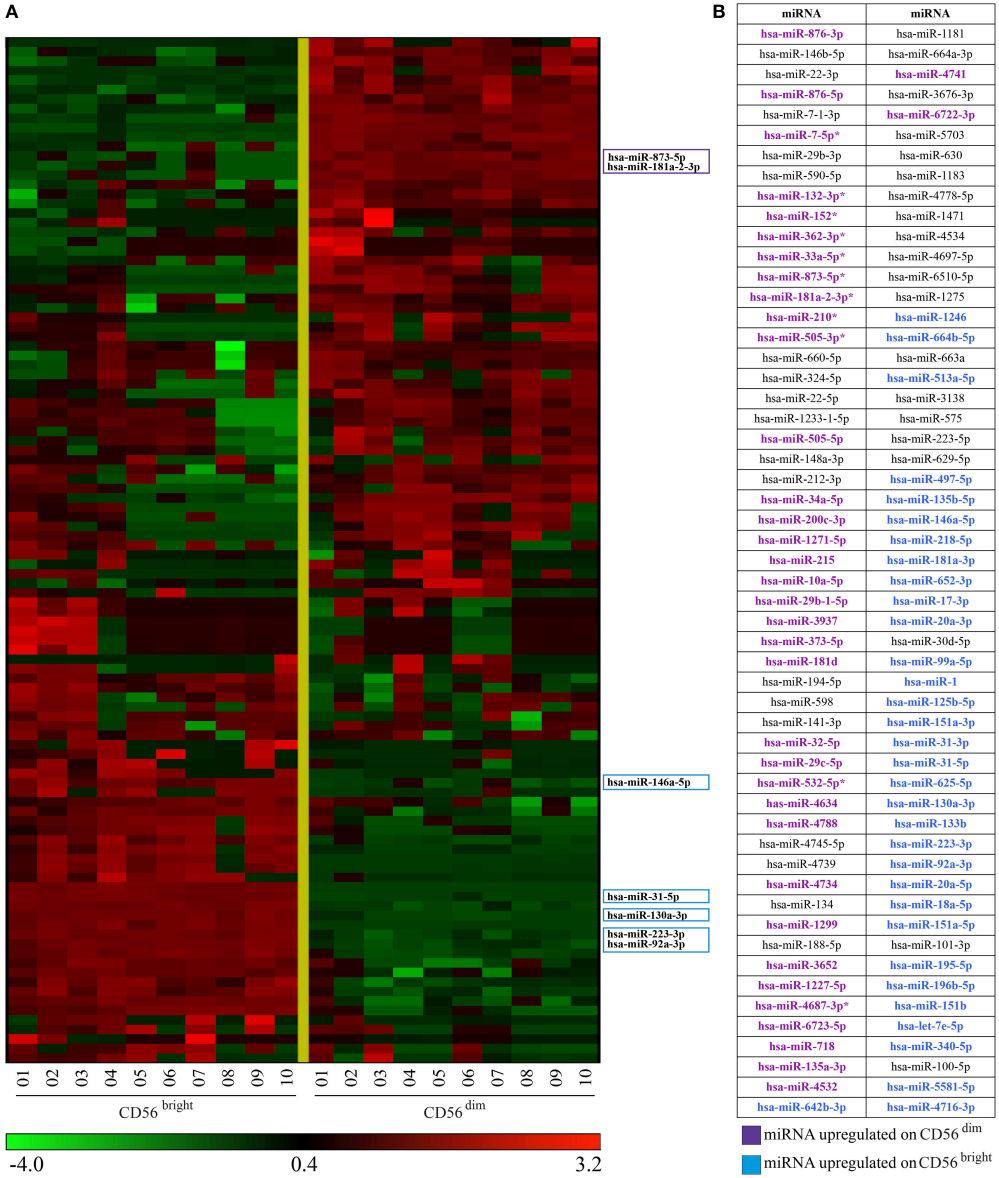
Heatmap representing CD56^bright^/CD16^−^ and CD56^dim^/CD16^+^ NK cells miRNAs signature obtained with multivariate statistical method. **(A)** Heatmap of the 108 miRNAs signature identified by the l1l2-penalized regularization method. Colored boxes indicate 7 of the most regulated miRNAs. **(B)** miRNAs listed in **(A)** (from top to bottom) are indicated. Color legend: the most up-regulated miRNAs on CD56^bright^/CD16^−^ are indicated in light blue and the most up-regulated miRNAs on CD56^dim^/CD16^+^ NK cells are indicated in purple.

The list obtained from the multivariate analysis includes all the miRNAs found with the univariate analysis (37 miRNAs, Figure 2) and 71 additional miRNAs identified only by this method. Figure 3 shows the heatmap of the 108 miRNAs signature identified (Figure 3A) and the names of the miRNAs listed in the order used in the heatmap (Figure 3B). The result was a miRNA signature associated with a perfect prediction accuracy (100%, corresponding to a zero error score).

### miRNA qPCR validation of univariate analysis

**Figure 4:**
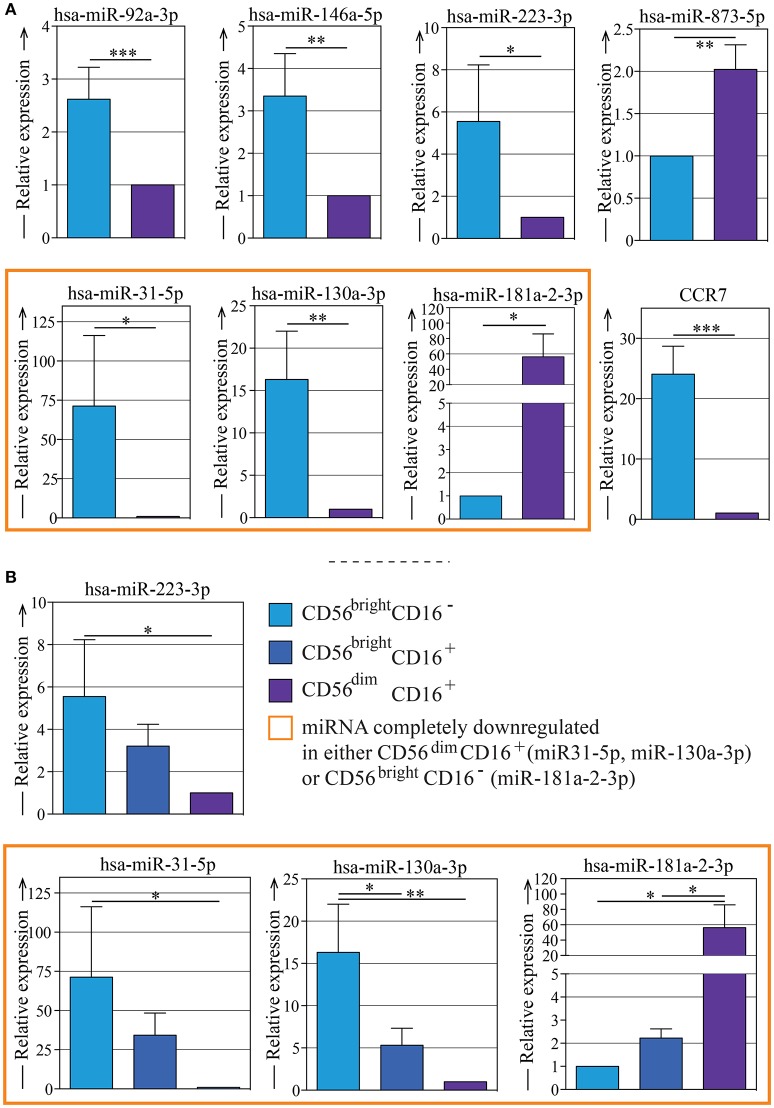
Quantitative RT-PCR validation of the expression of seven of the most regulated miRNAs on different NK subsets. **(A)** Quantitative RT-PCR validation of the expression of miR-92a-3p, miR-146a-5p, miR-223-3p, miR-873-5p, miR-31-5p, miR-130a-3p, and miR-181a-2-3p in CD56^bright^/CD16^−^ and CD56^dim^/CD16^+^ NK cell subsets. The qRT–PCR experiment was performed using samples prepared separately from the microarray experiment. Quantitative evaluation of CCR7 mRNA transcripts in human CD56^bright^ and CD56^dim^ NK cells by real-time RT-PCR was used as control. Data represents the mean ± SEM, *n* = 3 donors. *T*-test analysis comparing CD56^bright^ CD16^−^ and CD56^dim^ CD16^+^ NK cell subsets indicated as *p < 0.05, **p < 0.01, *** *p* < 0.001. **(B)** Quantitative RT-PCR validation of the expression of miR-223-3p, miR-31-5p, miR-130a-3p, and miR-181a-2-3p in CD56^bright^/CD16^−^, CD56^bright^/CD16^+^, and CD56^dim^/CD16^+^ NK cell subsets. The qRT–PCR experiment was performed using samples prepared separately from the microarray experiment. Data represent the mean ± SEM, *n* = 3 donors. One way Anova with Bonferroni's post-test comparing the three NK cell subsets indicated as *p < 0.05, **p < 0.01. Color legend: CD56^bright^/CD16^−^ NK cells are indicated in light blue, CD56^bright^/CD16^+^ NK cells are indicated in dark blue, CD56^dim^/CD16^+^ NK cells are indicated in purple,and the miRNAs that can be considered completely down-regulated in either CD56^bright^/CD16^−^ or CD56^dim^/CD16^+^ are indicated with an orange frame.

We selected 7 of the most regulated miRNAs (indicated in Figures 2, 3) that were differently expressed in CD56^bright^ and CD56^dim^ NK cell subsets and we validated them by quantitative Real Time Polymerase Chain Reaction (RT-PCR). The purity of the two NK cell subsets was confirmed by the gene expression analysis of CCR7 (a marker selectively expressed in CD56^bright^ cells subset but not expressed in CD56^dim^ NK cell subset as shown in Figure 1C). These analyzes confirmed once again the levels of expression of these miRNAs in the two NK cell subsets (Figure 4A).

Then we wondered if the miRNA signature found in the two main NK cell subsets (CD56^bright^ and CD56^dim^) could be linked to the sequential steps of maturation. Thus, we sorted the additional NK cell subset CD56^bright^ CD16^+^ (Supplementary Figure 1), that is known to represent an intermediate step of peripheral blood-NK cell maturation, and performed qPCR analysis by looking at the most differentially expressed miRNAs (hsa-miR-130a-3p, hsa-miR-31-5p, hsa-miR-181a-2-3p, hsa-miR-223-3p) (Figure 4B). We showed that the expression levels of these miRNAs on the CD56^bright^/CD16^+^ NK cell subset were halfway between those observed on the CD56^bright^/CD16^−^ and those on the CD56^dim^/CD16^+^ NK cell subsets. In particular, by comparing the qRT-PCR data obtained from CD56^bright^/CD16^−^, CD56bright/CD16^+^ and CD56^dim^/CD16^+^ NK cells subsets, the existence of a significant progressive change in these miRNAs expression was revealed: this detail represents the trend of NK cell subsets maturation that shows how the expression reaches different levels as the cells goes on maturing. (Figures 4A,B).

In addition, we also sorted the CD56^dim^/CD57^+^ and the CD56^dim^/CD57^−^ NK cell subsets, recognized as the most mature NK cell subsets. We found that hsa-miR-130 and hsa-miR-31-5p as well as hsa-miR-181a-2-3p were expressed at similar levels in CD56^dim^/CD57^+^and CD56^dul^/CD57^−^ NK cell subsets (data not shown). This supports the hypothesis that such miRNAs are important molecules for the switch between CD56^bright^ and CD56^dim^ phenotypes but not for the terminal differentiation that ends with a CD56^dim^/CD57^+^ phenotype.

### miRNA validation of multivariate analysis

The miRNA multivariate analysis, as reported in the sections above, identified a signature of 108 predictive miRNAs. In order to validate data derived by multivariate analysis, we selected 12 miRNAs composed of 6 miRNAs validated by univariate analysis (hsa-miR-223-3p, hsa-miR-92a-3p, hsa-miR-31-5p, hsa-miR-181a-2-3p, hsa-miR-130a-3p, hsa-miR-146a-5p) and additional 6 miRNAs identified by multivariate analysis which showed a two-fold change listed in Supplementary Table 1: hsa-miR-215-5p, hsa-miR-513a-5p, hsa-miR-532-5p, hsa-miR-3652, miR-6723-5p, hsa-miR-181a-3p. This filtering procedure was undoubtedly necessary to achieve a more compact and interpretable signature, but it induced an additional verification phase on the multivariate model. In fact, before proceeding with the validation, we had to make sure that a multivariate model based only on the 12 miRNAs of the filtered signature was still capable of a good prediction performance in discriminating CD56^bright^ and CD56^dim^. Therefore, we considered the original dataset limited to the 12 selected miRNAs, obtaining a shortened dataset of 20 samples and 12 variables. Using regularized least squares in a Monte-Carlo Cross Validation setting as in Barbieri et al. (37), we estimated a new predictive model that was able to achieve a 99.7% average prediction accuracy and associated with a *p*-value as low as 1.020e-20 from the two samples Kolmogorov-Smirnov test (Supplementary Figure 2B). This result showed that the 12 miRNAs model is an excellent classifier of CD56^bright^ and CD56^dim^ when considering miRNAs expressions measured with the microarray technology.

We then proceeded with a further validation step with the aim of verifying if such model was still capable of good prediction on new samples measured with a different technology, i.e., qPCR.

This is a very strong requirement on the generalization properties of the model, as the test set was completely independent from the learning set, having been acquired with a different technique and on different samples. To this aim, the expression of the 12 miRNAs was measured on 6 new samples (Supplementary Figure 3). We used the same statistical classifier to predict the status (CD56^bright^ or CD56^dim^) of the 6 samples measured with qPCR obtaining a correct prediction of 4 out of 6 samples. The entire validation procedure is depicted in detail in Supplementary Figure 2B.

### Target genes prediction

Since miRNAs function mainly through the inhibition of target genes, we next tried to identify the mRNA targets regulated by miRNAs differentially expressed in human NK cell subsets that might be involved in their function/maturation.

We considered six prediction tools for mRNA target genes prediction: TargetScan (38), Miranda (39), MirDB (40), Eimmo (41), Pictar (42), and PITA (31). Each tool was used to predict the target genes regulated by miRNAs that belongs to Supplementary Table 1, which contains 37 selected miRNAs up-regulated on CD56^dim^ and 33 miRNAs up-regulated on CD56^bright^, on the basis of multivariate analysis. A complete list of the potential target genes predicted by bioinformatics platforms is shown in Supplementary File 2 both for CD56^dim^ and CD56^bright^.

Since one of the goals of this study was the identification of novel miRNAs that may contribute to the NK cell development and differentiation, we focused our attention on potential target genes encoding for markers that are differently expressed on the two main NK cell subsets. These analyses identified some miRNAs differently expressed on CD56^bright^ compared to CD56^dim^ NK cells as potential regulator of targets genes encoding molecules of our interest (Table 1).

**Table 1:** List of some relevant NK surface marker genes targeted by specific miRNAs regulated in CD56^dim^ and CD56^bright^.

**List of some relevant NK surface marker genes targeted by specific miRNAs regulated in CD56**^**dim**^ **and CD56**^**bright**^		
**Gene Symbol**	**miRNA upregulated on CD56**^dim^	**miRNA upregulated on CD56**^bright^
CCR7	miR-32-5p-miR-1299	miR-1-let-7e-5p-miR-125b-5p-miR-218-5p
CMKLR1	miR-7-5p-miR-32-5p-miR-373-5p-**miR-873-5p**	miR-1-miR-17-3p-miR-18a-5p-miR-20a-3p-miR-20a-5p miR-31-3p-**miR-31-5p**-miR-218-5p-**miR-223-3p**
CX3CR1	miR-7-5p-miR-32-5p-miR-33a-5p-miR-34a-5p miR-132-3p-miR-362-3p-**miR-873-5p**	miR-1-miR-17-3p-miR-20a-3p-miR-20a-5p-miR-31-3p miR-31-5p-**miR-223-3p-**miR-340-5p-miR-497-5p
CXCR1	miR-32-5p-miR-34a-5p-miR-135a-3p-miR-362-3p **miR-873-5p-**miR-876-5p	miR-1-miR-31-3p-**miR-31-5p-**miR-135b-5p-miR-195-5p
FCGR3A	miR-7-5p-miR-10a-5p-miR-32-5p-miR-135a-3p miR-181d	miR-1-miR-133b-miR-135b-5p-miR-181a-3p miR-218-5p
GRZB		miR-1
IL2RA	**miR-873-5p-**miR-1299	miR-1-**miR-130a-3p-**miR-340-5p
KIR2DL1	miR-34a-5p-miR-181d-miR-373-5p	miR-1-**miR-146a-5p-**miR-181a-3p
KIR2DL2	miR-34a-5p-miR-181d-miR-373-5p	miR-1-**miR-146a-5p-**miR-181a-3p
KIR2DL3	miR-34a-5p-miR-181d-miR-373-5p	miR-1-**miR-146a-5p-**miR-181a-3p
KIR2DL4	miR-29c-5p-miR-34a-5p-miR-135a-3p-miR-210 miR-497-5p	miR-1-miR-135b-5p-**miR-146a-5p-**miR-195-5p
KIR2DL5A	miR-34a-5p	miR-1
KIR2DL5A	miR-34a-5p	miR-1
KIR2DL5B	miR-34a-5p	miR-1
KIR2DS1		miR-1
KIR2DS2	miR-34a-5p-miR-373-5p	miR-1
KIR2DS3	miR-34a-5p-miR-373-5p	miR-1
KIR2DS4	miR-29c-5p-miR-34a-5p-miR-373-5p	miR-1-miR-133b-miR-135b-5p-miR-195-5p-miR-497-5p
KIR2DS5	miR-34a-5p	miR-1
KIR3DL1	miR-34a-5p	miR-1-**miR-146a-5p**
KIR3DL2	miR-34a-5p	miR-1-miR-340-5p
KIR3DL3	miR-34a-5p	miR-1
KIR3DP1	**miR-873-5p**	**miR-146a-5p**
KIR3DS1	miR-34a-5p-miR-181d-miR-373-5p	miR-1-miR-135b-5p-**miR-146a-5p-**miR-181a-3p
KLRC1		miR-1
LILRB1		miR-1-miR-17-3p-miR-20a-3p-miR-20a-5p
NCAM1	miR-10a-5p-miR-29c-5p-miR-32-5p-miR-33a-5p miR-135a-3p-miR-152-miR-215-miR-200c-3p miR-210-miR-362-3p-miR-505-3p-miR-505-5p **miR-873-5p-**miR-876-3p-miR-876-5p-miR-1271-5p miR-4687 miR-513a-5p	3p-miR-1-miR-17-3p-miR-18a-5p-miR-20a-3p-miR-20a-5p miR-31-3p-miR-125b-5p-**miR-146a-5p-**miR-340-5p
NCR1		miR-1
NCR2	miR-132-3p-miR-181d	miR-1-miR-181a-3p
NCR3	miR-32-5p	
PDCD1	miR-32-5p-**miR-873-5p**	miR-1
PRF1	miR-7-5p-miR-32-5p-miR-132-3p	miR-1-**miR-146a-5p**

*Target genes for the 70 miRNAs included in this table were predicted using **six** prediction tools for mRNA target genes prediction (TargetScan, Miranda, MirDB, Eimmo, Pictar, and PITA). The table shows a selection of some of the most relevant NK cell surface marker genes with the corresponding miRNAs. The miRNAs are listed on the basis of their regulation on CD56^dim^ (left) and CD56^bright^ (right). Seven of the most regulated miRNAs are indicated in bold*.

In particular, our analysis identified hsa-miR-146a-5p as a possible regulator of targets genes encoding for different members of the most important HLA-I specific NK receptors, the KIR family (12). Notably, hsa-miR-146a-5p seems to be mainly involved in the regulation of inhibitory KIR expression (with the exception of KIR3DL2/3), while other miRNAs appear to be involved in the regulation of activating KIRs. The only exception is represented by KIR3DS1 that again appears to be regulated by hsa-miR-146a-5p (Table 1). In addition, one of the predicted target of hsa-miR-146a-5p is perforin mRNA.

It is important to remember that KIR receptors are selectively expressed by the CD56^dim^ NK cell population and absent on the CD56^bright^ cell population [with the exception of KIR2DL4 (15)] and that their expression increases according to the maturation state of NK cells. Furthermore, perforin expression is higher in CD56^dim^ NK cell compared to CD56^bright^ cell. In this context, the up-regulation of hsa-miR-146a-5p detected on CD56^bright^ NK cells may be in line with the down-regulation of KIRs and perforin expression on this subset.

To demonstrate that the KIR genes may represent targets for hsa-miR-146a-5p, we performed additional experiments focusing our attention on genes encoding the inhibitory KIR2DL1 and KIR2DL2 receptors. These KIR receptors exhibit complementary features to enable recognition of both HLA-C1 and HLA-C2 subtypes, the dominant KIR ligands.

### Dual-luciferase reporter assay to validate KIR2DL1 and KIR2DL2 as targets of miR-146a-5p

**Figure 5:**
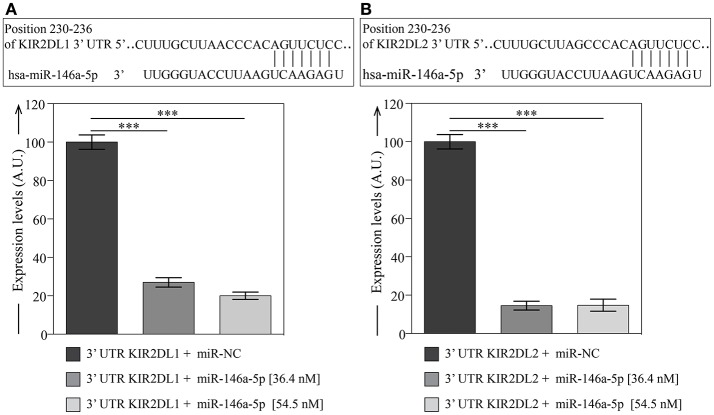
Dual-luciferase reporter assay validates KIR2DL1 and KIR2DL2 as targets of miR-146a-5p. miRNA binding sites predicted by Miranda and TargetScan within 3′UTR of KIR2DL1 **(A)** and KIR2DL2 **(B)** target genes and validation of miRNA binding. Columns represent the luciferase activity of 3′UTR plasmids containing 3′UTR of KIR2DL1 or KIR2DL2 with miR-146a-5p, relative to transfection with the same constructs and miR-NC. Relative luciferase expression (firefly normalized to Renilla) values of hsa-miR-146a-5p were normalized to miR-NC transfected controls. Data represents the mean ± SEM, *n* = 3 independent experiments. One way Anova with Bonferroni's post-test comparing 3′UTR plasmids with miR-146a-5p to miR-NC indicated as ***p < 0.001.

By using different target prediction software we predicted that hsa-miR-146a-5p has conserved binding sites in the 3′UTR of KIR2DL1 (Figure 5A) and KIR2DL2 (Figure 5B). To functionally verify the putative miRNA-mRNA interaction, we used plasmids containing the entire 3′UTR of both KIR2DL1 and KIR2DL2 and luciferase and Renilla reporter genes. COS-7 cells were transfected with these plasmids with hsa-miR-146a-5p or with negative control (miR-NC). Luciferase results revealed significant inhibition of luciferase activity for hsa-miR-146a-5p with both 3′UTR sequences of KIR2DL1 (Figure 5A) and KIR2DL2 (Figure 5B). Thus, we validated KIR2DL1 and KIR2DL2 as potential hsa-miR-146a-5p targets by luciferase reporter assay.

### *In silico* functional characterization of the predicted gene targets

In order to explore which pathways characterize the predicted gene targets, we performed a *in silico* functional characterization using the webtoolkit WebGestalt (43) (see Materials and Methods). In particular, we made the enrichment analysis considering the Kyoto Encyclopedia of Genes and Genomes (KEGG) (44).

**Figure 6:**
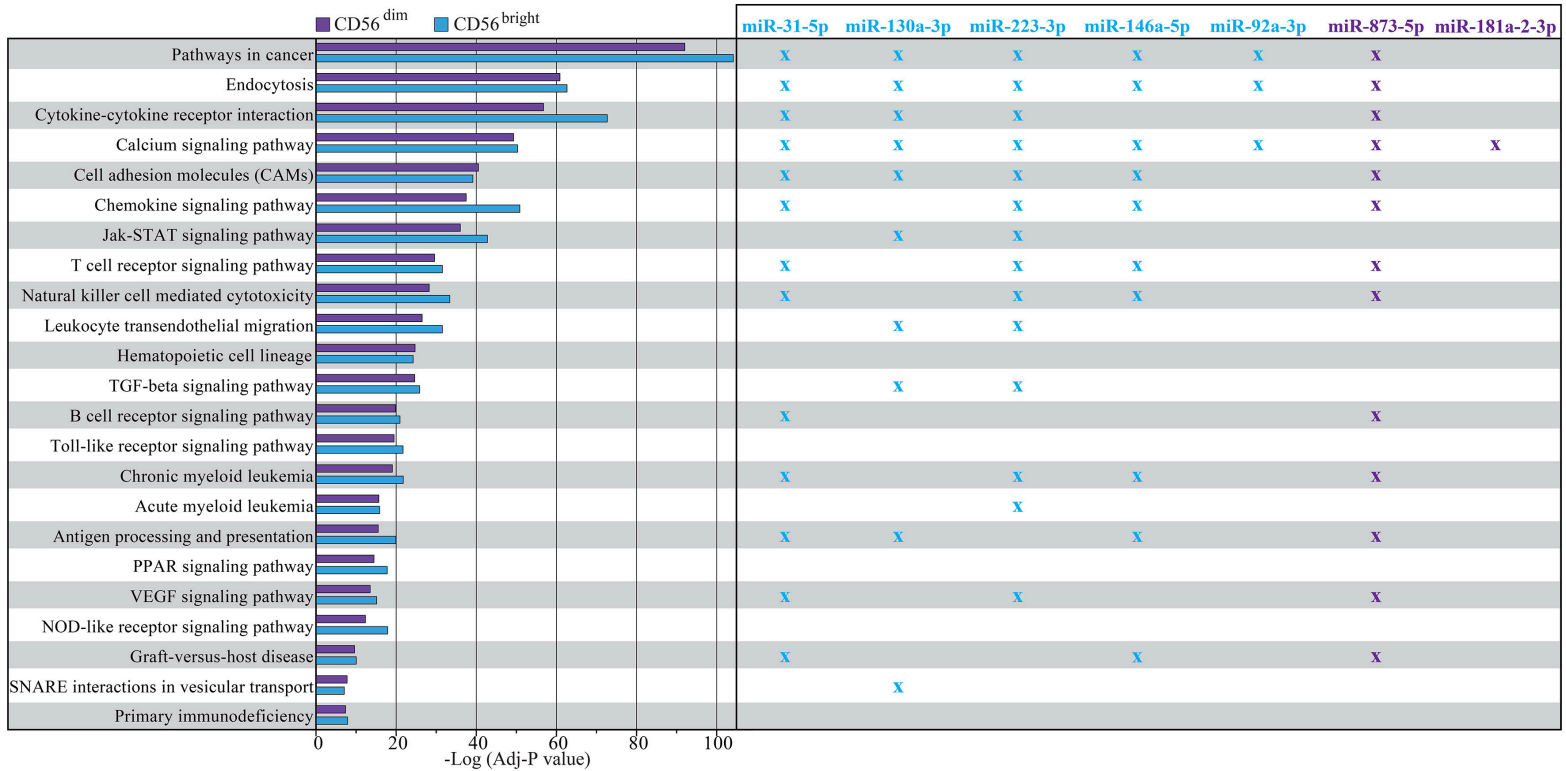
Enrichment analysis result using WebGestalt toolkit within KEGG database. The plot shows a selection of KEGG pathways shared by both CD56^dim^ and CD56^bright^ NK cells. The pathways are sorted according to a score corresponding to the log transformed average adjusted *p*-value. Higher scores correspond to a lowest average adjusted *p*-value. Crosses indicate in which pathways the putative targets of the most differentially expressed miRNAs are involved with an adjusted *p* > 0.05. Color legend: *P*-value of the pathway corresponding to CD56^dim^/CD16^+^ NK cell subset is indicated in purple, *P*-value of the pathway corresponding to CD56^bright^/CD16^−^ NK cell subset is indicated in light blue; miRNAs up-regulated on CD56^bright^/CD16^−^ NK cell subset and corresponding crosses are indicated in light blue, miRNAs up-regulated on CD56^dim^/CD16^+^ NK cell subset and corresponding crosses are indicated in purple.

In details we found 195 common KEGG pathways, 5 pathways specific for CD56^bright^ and 2 pathways specific for CD56^dim^ (Supplementary File 3). In Figure 6 we reported a selection of the common KEGG pathways that we considered majorly linked to phenotypic and functional features of NK cells indicating which of the validated miRNAs appear to be directly involved.

**Figure 7:**
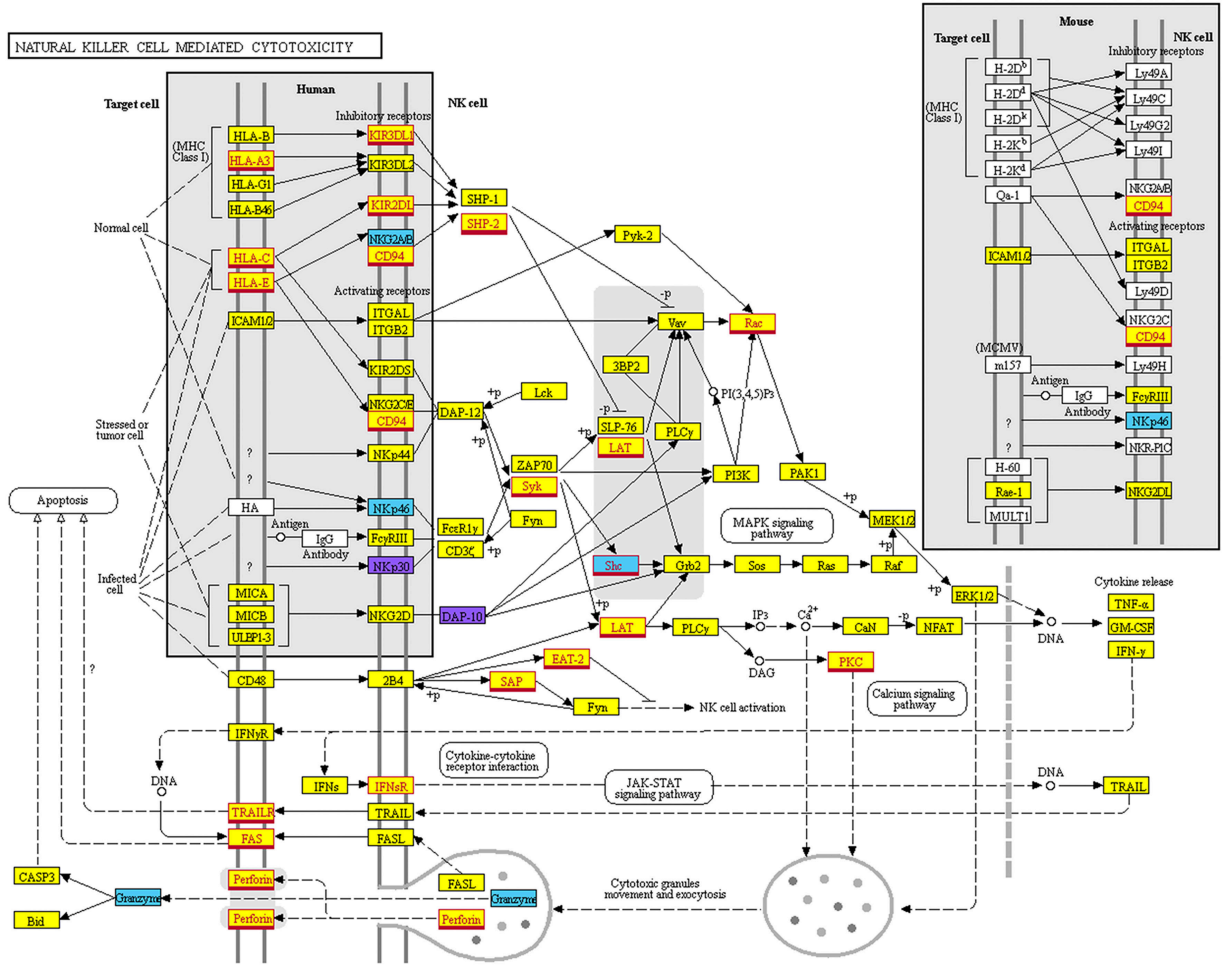
Natural Killer cell mediated cytotoxicity KEGG map. Map of the “Natural Killer cell mediated cytotoxicity” pathway derived from KEGG (hsa04650). Color legend: genes targeted only by miRNAs overexpressed on CD56^bright^ are indicated in light blue; genes targeted only by miRNAs overexpressed on CD56^dim^ are indicated in purple; genes targeted by miRNAs overexpressed on both CD56^bright^ and CD56^dim^ are indicated in yellow. Genes targeted by miR146a-5p are indicated with a red outline and font.

Among these KEGG-Pathways, we show, in particular, the “Natural Killer cell mediated cytotoxicity” pathway (Figure 7), where multiple putative targets of hsa-miR-146a-5p are involved, such as CD94, HLA-C, HLA-E, Perforin, and KIRs (including KIR2DL1/L2 that we validated as targets for this miRNA).

## Discussion

Recent studies regarding the development of human NK cell subsets showed that there is a continuum in NK-cell differentiation *in vivo* from a CD56^bright^/CD16^−^ to CD56^dim^/CD16^+^ phenotype. This was demonstrated by the detection of “intermediary” NK cell subsets, including the CD56^bright^/CD16^+^ NK cells that display intermediate characteristics typical of CD56^bright^ or CD56^dim^ cells. In addition, some data would suggest that CD56^dim^ cells may change their phenotypic features continuing to differentiate throughout their lifespan (8, 26–29). The loss of NKG2A and the acquisition of KIRs and CD57, together with some important functional changes, allow the definition of sequential steps of NK cell maturation (8, 29). Several intermediates of this maturation process are detectable in varying proportions in healthy donors. Importantly, functional, and phenotypic perturbation of the NK cell compartment and an unusual/aberrant redistribution of NK cell subsets has been found in several pathological conditions, such as inborn errors of immunity, severe chronic viral infections, and tumors (9, 45). In this context, the CD56^dim^NKG2A^−^KIR^+^LIR-1^+^CD57^+^ NK cell subset, when derived from individuals previously exposed to pathogens, particularly HCMV, may include “memory-like” NK cells (that display certain functional characteristics reminiscent of adaptive immunity cells) (7) or PD-1^+^ NK cells (characterized by high surface expression of the PD-1 inhibitory checkpoint, and by impaired anti-tumor activity) (46). However, understanding of the molecular mechanisms that define NK cell subsets development and function is largely incomplete.

Recently, miRNAs have been described as critical players in the post-transcriptional regulation of gene expression, thereby controlling many physiological processes including the development of several immune cell lineages. Moreover, some miRNAs have been shown to regulate both NK cell development and function (30, 32, 33).

**Figure 8:**
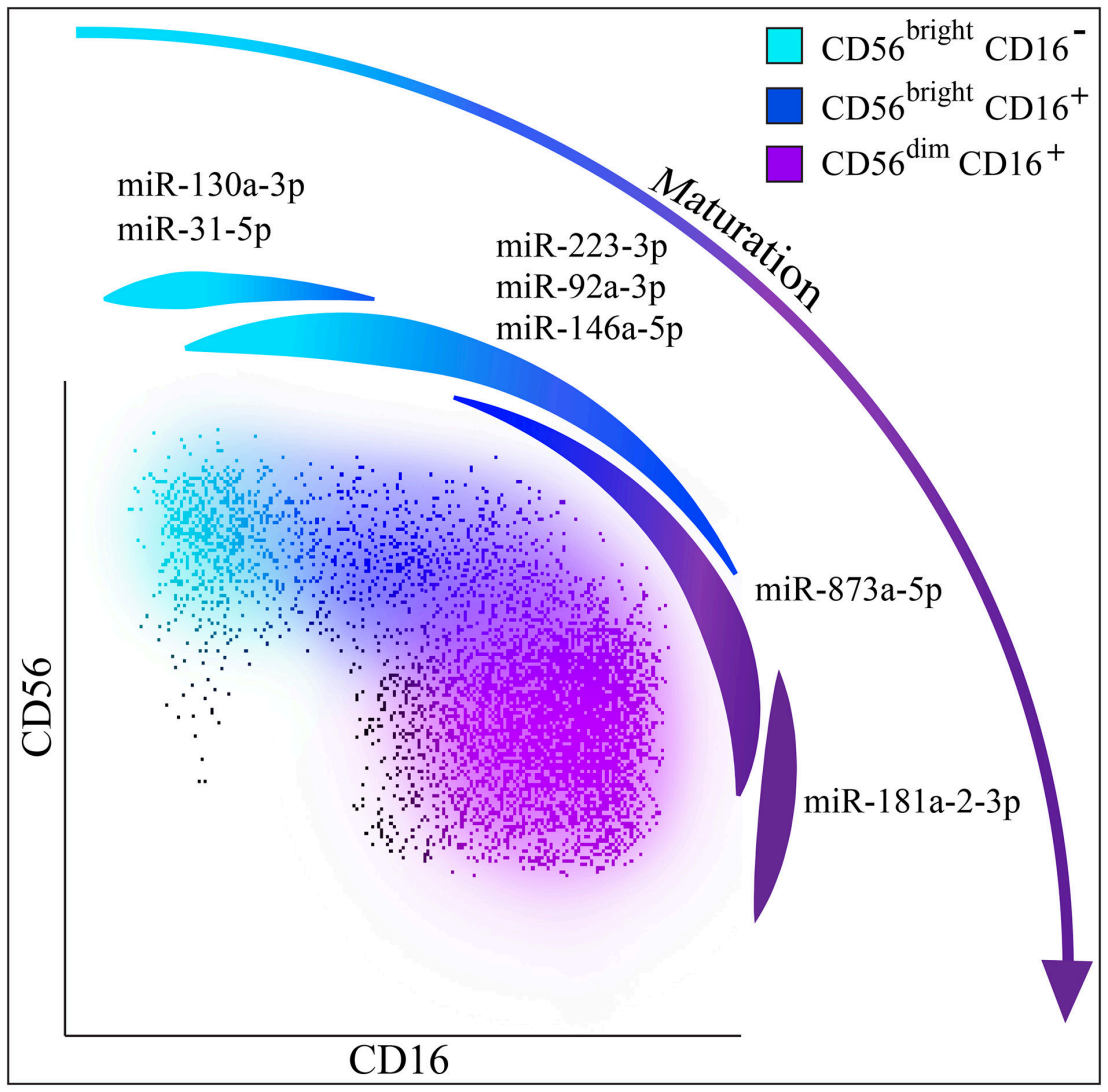
NK cells maturation and miRNAs. Hypothesis of NK cell maturation supported by differential miRNA expression in the different NK cell subsets. Color legend: CD56^bright^/CD16^−^ NK cells are indicated in light blue, CD56^bright^/CD16^+^ NK cells are indicated in dark blue and CD56^dim^/CD16^+^ NK cells are indicated in purple. Spot representing miR-130a-3p and miR-31-5p that can be considered exclusively expressed by CD56^bright^ NK cells is indicated in light blue; spot representing miR-223-3p, miR-92a-3p, miR-146a-5p up-regulated on CD56^bright^ NK cells is indicated in light blue/dark blue; spot representing miR-873a-5p up-regulated on CD56^dim^ NK cells is indicated in dark blue/purple and spot representing miR-181a-2-3p exclusively expressed by CD56^dim^ NK cells is indicated in purple.

In order to explore whether unique miRNA profiling may characterize NK cells with different phenotypic and functional features, we comprehensively analyzed the functional miRNA signatures of different NK cell subsets isolated from PBMC of healthy donors. We could detect distinct miRNA profiles in CD56^bright^ and CD56^dim^ NK cells, including unique miRNA signatures for each subset. Among the most regulated miRNAs, three miRNAs were strongly down-regulated (hsa-miR-146a-5p, hsa-miR-92a-3p, hsa-miR-223-3p), and one miRNA deeply up-regulated (hsa-miR-873-5p) in CD56^dim^ samples as compared to CD56^bright^ ones. Moreover, we identified miRNAs that are exclusively expressed by CD56^bright^/CD16^−^ NK cells (such as hsa-miR-31a-5p, hsa-miR-130a-5p) or by CD56^dim^/CD16^+^ NK cells (hsa-miR-181a-2-3p). Notably, these miRNAs display an intermediate level of expression on CD56^bright^/CD16^dim^ NK cells. The miRNA signature identified in our study further support the concept that CD56^bright^/CD16^−^ NK cells progress toward CD56^dim^ NK cells through an intermediate step represented by CD56^bright^/CD16^dim^ NK cells (Figure 8).

To the best of our knowledge, this is the first attempt to identify a miRNAs signature capable of precisely discriminating CD56^bright^ and CD56^dim^ NK cell subsets independently from the analysis of their surface phenotype. The fact that this signature may be applied to all healthy individuals, independently from their physiological heterogeneity, makes our results even stronger. In this regard, it is important to remember that results published to date are frequently based on analyses of single donors. In addition, our data were validated not only by a univariate analysis but also by means of a multivariate analysis approach that achieved 100% average prediction accuracy in discriminating CD56^dim^ from CD56^bright^ NK cells. The validation phase was completed by testing the multivariate model on qPCR data obtained by a different set of samples. Also this analysis achieved a good prediction performance.

The important role of miRNAs in regulating the NK cell function was confirmed by disrupting miRNAs in mouse NK cells (33). Interestingly, some recent data support a possible role of the miRNAs identified in the present work in the functioning of NK cells. For instance, miR-146a negatively regulates NK cell function and its silencing has been proposed as a therapeutic tool with a relevant potential to ameliorate the NK cell activities in liver diseases (47). miR-223 expression is involved in the hematopoietic cell differentiation. In particular, its expression increases as haematopoietic progenitor cells differentiate into red blood cells or NK cells, while a decrease was detected in cells differentiating toward neutrophils, monocytes, megakaryocytes and eosinophils (48). Interestingly, Granzyme B was reported among the validated target of miR-223 in mouse (49). However, both our target gene prediction and reports from other groups failed to confirm this data in humans (48).

Other miRNAs involved in hematopoietic cell differentiation belong to miR-181 family. All miR-181 contribute to the development of all three lymphoid lineages and are involved in human NK cell development by regulating Notch signaling (50). In addition, miR-223, miR-130a, and miR-181 families are deregulated during inflammatory disorders and cancer. In this context, miR-181a expression is up-regulated in haemopoietic malignancies and in hepatic and breast carcinomas (51). Recent data have also revealed that miR-130a is involved in the regulation of the activity of Nuclear factor-κB (NF-κB), one of the most important nuclear transcription factors involved in tumorigenesis. In particular, a negative feedback regulation of NF-κB/miR-130a/TNF-α/NF-κB has been described in cervical cancer cells (52). Finally, some of these miRNAs allow discrimination between different tumor types possibly representing a new class of blood biomarkers (53, 54).

In the present study, we also performed a prediction analysis of putative mRNA targets. This analysis allowed us to identify miR-146a-5p as a relevant regulator of inhibitory KIR expression in NK cells. KIRs play a crucial role in human NK cell development and function (12, 14, 55). Notably, *KIR* gene family is absent in rodents and is present only in primates. Thus, *KIR* genes were originated only recently, undergoing a rapid evolution.

The KIR gene family currently consists of 13 KIR genes (KIR2DL1, KIR2DL2/L3, KIR2DL4, KIR2DL5A, KIR2DL5B, KIR2DS1, KIR2DS2, KIR2DS3, KIR2DS4, KIR2DS5, KIR3DL1/S1, KIR3DL2, and KIR3DL3) and two pseudogenes (KIR2DP1 and KIR3DP1) encoded within the Leukocyte Receptor Complex (LRC) located on chromosome 19 (55). Upon recognition of specific HLA-class I ligands, inhibitory KIRs induce signals that block NK cell function. Individual KIRs bind distinct subgroups of HLA-class I allotypes, and are clonally expressed in NK cells, thus generating a repertoire of NK cells specific for different HLA-class I molecules. Dominant KIR ligands are HLA-C allotypes. On the basis of simple diallelic polimorphisms at position 77 and 80 of the heavy chain, HLA-C allotypes can be distinguished in two groups. Those with asparagine 77 and lysine 80 interact with the two Ig-like domains KIR2DL1, while those with serine 77 and asparagine 80 interact with KIR2DL2.

Recently, it has been shown that miR-146a-5p is involved both in tuning the activity, intensity, and duration of inflammation and in regulation of innate and adaptive immune responses with implication in tumor progression, and virus infection (56–58). In addition, miR-146a was shown to act as a negative regulator of T (59) and NK cell function *via* STAT1 signaling (47). Moreover, it negatively regulates IFN-γ production in super activated NK cells by targeting IRAK1 and TRAF6, with subsequent inhibition of the NF-κB signaling cascade (60).

In the present study, we predicted that hsa-miR-146a-5p has conserved binding sites in the 3′UTR of KIR2DL1 and KIR2DL2. Indeed, we could functionally verify and validate this putative miRNA-mRNA interaction by using a dual-luciferase reporter assay. It is well known that the genetic modification of NK cells is notoriously difficult to be achieved. Researchers around the world are studying different approaches to improve these techniques. Some results have been obtained by viral transduction or electroporation by using NK cells lines or *ex vivo* expanded primary NK cells (61). However, no studies have been done on miRNAs transfection of different NK cell subsets. Remarkably, our luciferase reporter assay demonstrates that hsa-miR-146a-5p could interact with the 3′UTR of the KIR2DL1/KIR2DL2 mRNA, modulating its expression. Although these experiments were not performed on PBMC-derived NK cells, such miRNA-mRNA interplay is clearly in accord with our data indicating that hsa-miR-146a-5p and KIR expression was inversely correlated on NK cells.

Interestingly, hsa-miR-146a-5p shows some putative target sites in the 3′UTR of other KIRs and perforin (see Table 1). Based on these findings, it will be important to functionally validate these other putative interactions since both KIRs and perforin play critical role in the effector function of CD56^dim^ NK cell subset. In this context, additional miRNAs, such as miR-27a-5p, miR-378 and miR-30e, have been shown to interfere with NK cell cytolytic activity by targeting the granule proteins granzyme B and perforin (62).

Inhibitory KIRs play a central role in modulating NK effector function by preventing NK cell activation on binding with their ligands, primarily HLA-C molecules. KIR inhibition has been shown to be of great clinical relevance in the allogeneic haplo-mismatched stem cell transplantation (SCT) model. KIR/KIR-ligand mismatch between donor and recipient has been shown to be associated with lower rates of leukemia relapse and higher survival (63, 64), suggesting that, in the absence of KIR/KIR-ligand binding, “alloreactive” NK cells may eradicate residual leukemia cells. In light of these findings, to pharmacologically exploit NK alloreactivity, a fully human mAb anti-KIR 1-7F9 (IPH2101) was first generated, and then a recombinant version of this mAb was developed with a stabilized hinge region (Lirilumab) (65, 66). A number of evidences indicate that a high expression of KIR often correlates with poor prognosis in patients with different solid tumors. In addition, anti-KIR mAb increased the NK cell-mediated lysis of HLA-I^+^ tumor cells. Thus, it is conceivable that this immunotherapeutic approach may be soon extended to non-hematological tumors.

Recently, a novel NK cell subset referred to as “PD-1^+^ NK cells,” has been identified in HCMV^+^ individuals and in tumor patients (46). This subpopulation, is mainly composed of fully mature NK cells, expressing the KIR^+^ NKG2A^−^ CD57^+^ surface phenotype, and displays a reduced capacity to kill PD-L^+^ tumor cells. This impaired function is due to the surface expression of high levels of the inhibitory checkpoint PD-1 (46). Due to its ineffective ability to kill tumor cells, it is important to evaluate the conditions leading to the generation of these cells, in particular in patients with advanced cancers. In this context, our present study indicates that hsa-miR873-5p, one the the miRNAs up-regulated on CD56^dim^ NK cell subset, may be involved in the regulation of the surface expression of PD-1 (see Table 1) with important implications in the control of NK cell-mediated anti-tumor activity. In recent years, NK cells gained interest as a highly attractive tool for cancer immunotherapy. In this context, our results suggest to add hsa-miR-146a-5p to the list of regulators of NK cell function. Indeed, this miRNA may regulate the surface expression of KIR and, as a consequence, the NK cell mediated cytotoxicity. Progress in understanding the biology of NK cell subsets and of the mechanisms regulating the expression of receptors relevant for anti-tumor activity represents a critical key to better understand not only the process of NK cells maturation but also to allow NK cell manipulation to further improve their anti-tumor and anti-viral activity. Thus, although further investigations are required to better understand the role of hsa-miR-146a-5p and the mechanism regulating NK cell function, these results may provide an additional tool for a possible use of this miRNA in tumor therapy. Accordingly, determining the miRNA expression profile of NK cell subpopulations may provide information on the post-transcriptional regulation mechanisms controlling NK cell maturation and function. In conclusion, the present study provides the first evidence of the presence of differentially expressed miRNAs in human NK cell subsets, and offers valuable clues for further elucidation of miRNA-mediated regulation of NK cell maturation and function. In addition, it suggests new, mi-RNA-based, therapeutic approaches to unleash NK cell effector functions in the cure of cancer.

## Materials and methods

### Sample preparation

Buffy coats from healthy donors were obtained from the Immunohematology and Transfusion Center at the IRCCS San Martino Polyclinical Hospital, Genoa, Italy.

All subjects gave written informed consent in accordance with the Declaration of Helsinki. The protocol was approved by the Ethical committee of IRCCS San Martino Polyclinical Hospital, Genoa, Italy (39/2012).

Mononuclear cells from heparinized peripheral blood were obtained by density gradient centrifugation over Ficoll (Sigma, St. Louis, MO). Pure populations of NK cells were obtained from PBMC using the NK cell isolation kit (Miltenyi Biotec, Bergisch Gladbach, Germany) according to the manufacturer's instruction. In some experiments, MACS CD15 Micro beads were added to further improve depletion of granulocytes. The purity of NK cells was >98% NK cells (defined as CD56^+^/CD3^−^ CD19^−^/CD14^−^).

Freshly purified NK cells were then analyzed on BD-FACSAria II (BD-Biosciences) and sorted based on the expression of CD56 and CD16 molecules to collect the CD56^bright^/CD16^−^, CD56^bright^/CD16^dim^, and the CD56^dim^/CD16^+^ NK cell subsets separately (Figure 1 and Supplementary Figure 1). After each sorting, a complete phenotypic analysis of the populations collected was performed using the BD-FACSVerse (BD-Biosciences). Sorted cells were washed in PBS and the pellet freeze-dried (20 s in liquid nitrogen and then stored at −80°C).

### Flow cytometry analyses and monoclonal antibodies

The following purchased mAbs were used in this study: Anti-CD56-PC7 (clone c218), and anti-NKG2A allophycocyanin (Z199 clone) were purchased from Beckman Coulter/Immunotech (Marseille, France); anti-CD3-Viogreen (BW264/56 clone), anti-CD19-VioGreen (LT20 clone), anti-CD14-Viogreen (TÜK4 clone), and anti–KIR2DL1-S1 FITC (11PB6 clone), mAbs were purchased from Miltenyi Biotec (Bergisch Gladbach, Germany); anti-CD16–PerCP-Cy5.5 (3G8 clone), anti–KIR2DL2/L3/S2–FITC (CH-L clone) from BD Bioscience; Anti-hCCR7 (IgG2a, clone 150503) was from R&D Systems Inc (Abingdon, United Kingdom); anti-CD69 (FN50 clone, IgG1) were purchased from BioLegend (San Diego, Calif). The anti-KIR3DL1/L2/S1 mAb (AZ158 clone) was isolated in our laboratory and the specificity has been validated in the corresponding patent (WO2010081890A1). Cytofluorimetric analyses were performed on a FACSVerse (Becton Dickinson, Mountain View, Calif), and data were analyzed with FACSuite software version 1.0.3.

### RNA preparation

Total RNA including miRNAs was isolated using the miRNeasy Mini Kit (Qiagen). RNA samples were quality-checked via the Agilent 2100 Bioanalyzer platform (Agilent Technologies). RNA integrity (RIN) was determined by means of capillary electrophoresis by using a 2100 Bioanalyzer (Agilent Technologies, Santa Clara, USA). The threshold for RNA quality suitable for microarray analysis was considered to be a RIN>6.0 (67). All RNA samples used in the microarray assays and real-time PCR revealed RIN values between 7.6 and 9.4.

### Hybridization of human miRNA microarrays

Sample labeling and hybridization was performed according to the Agilent miRNA Complete Labeling and Hyb Kit (Agilent Technologies Santa Clara, USA) following the manufacturer's protocol. Briefly, 100 ng of total RNA was labeled using T4 RNA ligase, incorporating Cyanine 3-Cytidine biphosphate (pCp). The Cyanine-3-labeled miRNA samples were prepared for One-Color based hybridization with complete miRNA Labeling and Hyb Kit (Agilent Technologies, Santa Clara, USA) according to the manufacturer's instructions. Labeled miRNA samples were hybridized overnight (20 h, 55°C) to Agilent Human microRNA Microarrays 8x60K v19 using Agilent's recommended hybridization chamber and oven. Afterwards, microarrays were washed with increasing stringency using Gene Expression Wash Buffers (Agilent Technologies, Santa Clara, USA). Fluorescent signal intensities were detected with Agilent's Microarray Scanner System (Agilent Technologies) and extracted from the images using Feature Extraction 10.7.3.1 Software (Agilent Technologies, Santa Clara, USA). For determination of differential miRNA expression FES derived output data files were further analyzed using the GeneSpringGX software (Agilent Technologies).

### Univariate statistical analysis

The experimental design of this study contains groups of matched sample pairs [CD56^bright^ and CD56^dim^] obtained from 10 individuals. Therefore, statistical tests were performed to identify miRNAs differentially expressed between the sample groups.

The intensity data of the 10 individual microarrays were subjected to probe summarization, thresholding (1.0), and log_2_-transformation using GeneSpring v12.6 (Agilent Technologies). Subsequently, the arrays were normalized between each other by quantile normalization.

The miRNA data sets were normalized by quantile normalization and base-2 logarithms (log_2_) intensities were calculated. The variability between samples was visualized by centering the normalized data relative to the median of all samples for each miRNA. These centered data were calculated by subtracting the median of all samples from expression value of each individual. The recentered values are then represented in a two-color heatmap format on a log_2_ scale (see also the heatmap-coded columns median centered log_2_ intensities in the Supplementary File 1). The transformed log_2_ intensity values were further adjusted to reduce a batch effect. The corresponding batch-adjusted data are provided in the table “normalized log_2_ data bc” within the Supplementary File 1.

These normalized batch-corrected log_2_ intensity values were used in subsequent analyses to identify miRNAs differentially expressed between the CD56^bright^ and the CD56^dim^ NK cell populations. For the identification of differentially expressed miRNAs, the robustness of detection and the statistical significance were taken into account. With regard to statistical significance, we applied a standard selection criteria based on the following criteria: in the comparison of two groups, a miRNA was classified as induced if its Adj. *p*-value was ≤ 0.05 with difference in the median expression level (fold-change) of at least two-fold; a miRNA was considered repressed if its Adj. *p*-value was ≤ 0.05 with a fold-change value ≤ - 2.0 (Supplementary File 2).

The unsupervised hierarchical clustering was performed by using the online tool MORPHEUS (https://software.broadinstitute.org/morpheus/). Specifically, Figure 2 shows the results of the bi-clustering analysis of both miRNAs and NK cell samples.

The data presented here have been deposited in the National Center for Biotechnology Information (NCBI) Gene Expression Omnibus (GEO) with the GEO accession number GSE116743 [Internet address: (68)].

### Multivariate statistical analysis

In supervised multivariate statistics, the goal is to model the relationship between input data (samples) and their corresponding labels (cell type, bright, or dim) by looking for a function depending on several variables (miRNAs) at a time. Such a function should be able to: (i) have good prediction performance on the given data, (ii) generalize on previously unseen data, and (iii) effectively describe the interplay between the measured variables. The final outcome of a classifier is the prediction of labels associated to a set of input examples. To assess a prediction performance, one should define appropriate metric, such as the accuracy score. Accuracy is defined as the ratio of correctly predicted labels.

In this context, regularization methods are a popular class of machine learning techniques that can be expressed as the minimization problem of one *loss* function V that measures the adherence of the objective function to the data and one or more *regularization penalites* R that introduce additional information used to solve the problem: min_*f*_ *V*(*f*(*X*),*y*) +; λ*R*(*f*).

Different choices of V and R lead to different algorithms. The parameter λ controls the trade-off between the adherence of the model to the data and the regularity of the function *f*. Choosing the regularization parameter appropriately leads to unbiased statistical models (69). In the remainder of this section we will illustrate the two regularization methods used in the present work. The software library we used is implemented in Python and publicly available at https://github.com/slipguru/palladio.

#### Variable selection with sparse regularization

For multivariate variable selection, we chose l1l2, an embedded regularization method that combines two penalty terms, one enhancing sparsity (l1 norm) and the other retaining correlated variables (l2 norm). The algorithm can be tuned to give sets of discriminative variables of different sizes [see (70, 71) for a detailed description of the method]. Assume we are given a collection of n samples, each represented by a d-dimensional vector x of measurements (e.g., the miRNAs expression vector). Each sample is also associated with a binary label y, assigning it to a class (e.g., CD56^dim^ and CD56^bright^).

Therefore, this dataset is represented by a *n* × *d* matrix X and a n-dimensional labels vector y. Using only a subset of the given data (learning set), l1l2 looks for a linear model *f* (*x*) = β^*^x, whose sign gives the classification rule that can be used to associate a sample to one of the two classes. The classification performance of *f* (*x*) is then assessed on the remaining samples (test set) that were not used to build the model. The vector of weights β^*^ is forced to be a sparse vector, meaning that those variables which have zero value will not contribute in building the estimator *f* (*x*). The weight vector β^*^ is found in the so-called model selection phase, which consists in selecting the optimal values for the regularization parameters.

In order to guarantee an unbiased result, model selection and classification accuracy assessment are performed within two nested cross-validation loops, similarly to Barla et al. (72). As a consequence of the external loop of cross validation, l1l2 provides a set of B = 5 lists of discriminant variables (see Supplementary Figure 2A). Therefore, it is necessary to choose an appropriate criterion (72) in order to assess a common list of relevant variables. Our criterion is based on the absolute frequency, i.e., we decided to promote as relevant the most stable genes across the lists. The final lists of variables were chosen according to the slope variation of the number of selected genes vs. frequency, its value being 50%. The adoption of this approach let us to cut out those variables not stable across the cross-validation lists.

The l1l2 variable selection approach takes as input the entire dataset and, following a cross validation procedure, splits it B times in B different learning/test set pairs (blue/green rectangles). In our experiments B was chosen equal to 5. Each split is treated as an independent binary classification task where the two classes are CD56^bright^ and CD56^dim^, respectively. The goal is to find a sparse predictive model based on few relevant and predictive miRNAs out of all 2042 available miRNAs. Therefore, for each split, the l1l2 method learns from the learning set a statistical model via model selection and assesses its predictive performance on the test set. The result is the prediction error and the list of relevant variables for that particular split. Finally, the overall prediction error is evaluated by averaging all B = 5 errors. An aggregate list of relevant variables is also provided, ranked according to how many times each variable has been selected over the B = 5 splits.

#### Validation and robustness assessment

In the validation phase, we aimed at assessing if the linear model obtained with l1l2 was still achieving good prediction performance on a separate set of data from a different measuring technique. After selecting the 108 relevant and possibly correlated variables, a further selection step was performed, based on domain expertise and current literature. This led us to a signature of 12 miRNAs. The first step of our validation procedure consisted in assessing if the 12 miRNAs still retained the discriminative power of the larger set automatically selected by l1l2. To this aim, we used PALLADIO, a machine learning framework based on regularization methods. It provides, along with an assessment of prediction performance, an estimate of its reliability (37) by means of a non- parametric two samples Kolmogorov-Smirnov test (Supplementary Figure 2B).

### Microarray data validation by real-time PCR

The microarray data was validated by real-time PCR according to the manufacturer's instructions (Qiagen; miScript Reverse Transcription kit, catalog no. 218060; miScript Primer Assays for the following miRNAs: hsa-miR-92a-3p, catalog no. MS00006594, hsa-miR-146a-5p catalog no. MS00003535, hsa-miR-133b catalog no. MS00031430, hsa-miR-130a-3p catalog no. MS00003444, has-miR-31-5p catalog no. MS00003290, has-miR-223-3p catalog no. MS00003871, hsa-miR-181a-2-3p catalog no. MS00008834; hsa-miR-873-5p catalog no. MS00010605; hsa-miR-181a-3p catalog no. MS00006692; hsa-miR-215-5p catalog no. MS00003829; hsa-miR-513a-5p catalog no. MS00009912; hsa-miR-532-5p catalog no. MS00004571; hsa-miR-3652 catalog no. MS00023128; hsa-miR-6723-5p catalog no. MS00045794; miScript SYBR Green PCR kit, catalog no. 218073). Then, miRNA was normalized to the small nucleolar RNA RNU6B (primer assay, catalog no. MS00014000). The real-time PCR reactions were performed for target miRNAs and for the internal reference (U6), with each sample analyzed in triplicate. For qPCR data analysis, the ΔΔCt method, as previously described by Schmittgen and Livak (73), was used to calculate the relative expression levels of the target miRNAs. For cDNA synthesis we used Qiagen RT^2^ First Strand Kit (catalog no. 330401). RT^2^ PCR Assay Gene Expression was performed for CCR7 chemokine receptor 7 (NM_001838). The GAPDH was used for normalization in the fold change expression data calculations. After qPCR relative expression is determined with the ΔΔCt method as previously described.

### Target gene prediction tools

Several published target prediction algorithms have been used for the prediction of miRNA targets. Generally, such software mainly use sequence complementarity, evolutionary conservation among different species, and thermodynamic criteria to estimate the probability of a miRNA:mRNA duplex formation. For further reading, the review by Bartel published in 2009 (30) is recommended (http://www.ncbi.nlm.nih.gov/pubmed/19167326). For gene target prediction, we considered six prediction tools: miRanda [http://www.microrna.org (39)], mirDB [http://mirdb.org/miRDB/index.html (40)], Pictar [http://pictar.mdc-berlin.de/ [42]], PITA [http://genie.weizmann.ac.il/pubs/mir07 (31)]. TargetScan [http://targetscan.org (74)], ElMMo [http://www.mirz.unibas.ch/ElMMo2 (41)]. Concerning TargetScan we used the Predicted Targets (default predictions) file belonging to the 7.0 release. Concerning PITA we considered the PITA_sites_hg18_0_0_ALL file of the last release (number 6). Concerning PicTar we downloaded and used all the targets from doRiNA database (75), as suggested in the home page of this prediction tool. Concerning miRanda we considered the predictions from the August 2010 release. Specifically we used the most completed file that includes the “Non-good mirSVR Score and Non-conserved miRNAs.” Concerning mirDB we considered the last version (5.0). Concerning EImmo we used the last release, number 3, updated in January 2009.

### Luciferase assay

For this purpose, we used the pEZX-MT06 target reporter vectors containing the entire 3'UTR sequence for KIR2DL1 and KIR2DL2 (GenBank Accession: NM_014218.2, HmiT010083-MT06 from GeneCopoeia; GenBank Accession: NM_014219.2, HmiT010084-MT06 from GeneCopoeia) containing putative miR-146a-5p binding sites and transiently transfected them into COS-7 cells by using the Lipofectamin 2000 reagents according to the manufacturer recommendations (Invitrogen). COS-7 cells were grown up in 24-well plates to a confluency of about 80%, followed by a co-transfection of hsa-miR-146a-5p mimic (two different concentrations: 36.4 nM and 54 nM, Qiagen) together with 150 ng of each pEZX-MT06 constructs (HmiT010083-MT06 and HmiT010084-MT06). The luciferase activity was compared to values obtained after we co-transfected the negative control (miR-NC, Allstars negative control, Qiagen) and the plasmids (HmiT010083-MT06 and HmiT010084-MT06). Forty-eight hours after transfection, cells were lysed in 100 μl of passive lysis buffer according to the Luc-Pair Luciferase Assay Kit 2.0 (GeneCopoeia); 20 μl of the lysate was used for the luciferase activity measurements following the instructions of the Dual-Luciferase Reporter Assay System (Promega). Luciferase assays were run on a LUMIstar Luminometer (BMG Labtech) in three independent biological replicates.

### Functional characterization of the signature

For the functional analysis of the miRNA signature we used the on-line gene set analysis toolkit WebGestalt (43)^1^WEB-based GEne SeT AnaLysis Toolkit (WebGestalt): update 2013. Nucleic Acids Res, 41 (Web Server issue, W77-83). The toolkit performs the functional characterization by a gene set enrichment analysis in several databases including KEGG (44). Given a KEGG pathway and a reference set (such as the entire human genome) the enrichment is based on the comparison between the fraction of signature genes in the pathway and the fraction of pathway genes in the reference set. The signature is enriched in the KEGG pathway if the former is larger than the latter fraction. To perform the enrichment analysis in KEGG, we selected the WebGestalt human genome as reference set, *p* ≤ 0.05 as level of significance, 3 as the minimum number of genes, the Bonferroni correction to correct for multiple hypotheses and the default Hypergeometric test as statistical method.

One of the specific KEGG pathways we found enriched (NK cell mediated cytotoxicity, hsa04650) was colored using the “Color Pathway” tool available in KEGG Mapper that is a suite of mapping tools provided by KEGG (KEGG Copyright Permission 180201).

### Statistical analysis

Statistical analysis was performed with Graphpad Prism (La Jolla, CA) software. For statistical analysis of cytofluorimetric experiments (Figure 1C) and of RT-PCR data on CD56^bright^/CD16^−^, and CD56^dim^/CD16^+^ NK cells subsets (Figure 4A and Supplementary Figure 3) was used non-parametric t-test (Mann Whitney test). *P*-value of < 0.05 (^*^), < 0.01 (^**^), < 0.001 (^***^), and < 0.0001 (^****^) was considered statistically significant, when not indicated, data were not statistically significant. Multiple comparisons between RT-PCR data on CD56^bright^/CD16^−^, CD56^bright^/CD16^+^, and CD56^dim^CD16^+^ NK cells subsets were performed using univariate analysis of variance (One way Anova with Bonferroni's post-test; Figure 4B). Cytofluorimetric experiments are reported as the sample mean ± the standard deviation (SD) (Figure 1C) while RT-PCR data are reported as the sample mean ± the standard error of the mean (SEM) (Figures 4A,B and Supplementary Figure 3).

## Author contributions

SP, MS, PC, and MG designed, performed research, and interpreted data. FL performed sorting experiments. LM, AM, and SS interpreted data. EM, SC, and AB designed and performed research, interpreted data, and wrote the article.

### Conflict of interest statement

The authors declare that the research was conducted in the absence of any commercial or financial relationships that could be construed as a potential conflict of interest.

## Footnotes

^1^(http://bioinfo.vanderbilt.edu/webgestalt/)

## Abbreviations

NK: natural killer cells
KIRs: killer immunoglobulin-like receptors
HLA: human leukocyte antigen
NCRs: natural cytotoxicity receptors
mAb: monoclonal antibody
miRNAs: microRNAs
RT-PCR: real time polymerase chain reaction
PBMC: peripheral blood mononuclear cells.
