# Supplementary material for: New miRNA Signature Heralds Human NK Cell Subsets at Different Maturation Steps: Involvement of miR-146a-5p in the Regulation of KIR Expression
Source: Front Immunol. 2018 Oct 15;9:2360. doi: 10.3389/fimmu.2018.02360 (PMC6196268; doi:10.3389/fimmu.2018.02360)
Supplement: Supplementary file 1 [file Data_Sheet_1.ZIP › Supplementary File 1/Legend Supplementary File 1.docx]

**New miRNA signature heralds human NK cell subsets at different maturation steps: involvement of miR-146a-5p in the regulation of KIR expression**

**^1^Silvia Pesce^#^, ^2^Margherita Squillario^#^, ^1,3^Marco Greppi^#^, ^4^Fabrizio Loiacono, ^5^Lorenzo Moretta, ^1,3^Alessandro Moretta**^†^**, ^1,3^Simona Sivori, ^6^Patrizio Castagnola, ^2^Annalisa Barla*****, ^7^Simona Candiani*****, ^1,3^Emanuela Marcenaro***

^#^Equally contributed to this study

*These authors share senior authorship

^†^We dedicate this contribution to Alessandro Moretta who sadly passed away in mid February. We mourn his invaluable scientific insight and mentorship and, even more, his humanity, irony and smile.

^1^Department of Experimental Medicine (DIMES), University of Genoa, Genoa, Italy; ^2^Department of Informatic Bioengeneering, Robotic and System Engeneering, University of Genoa, Genoa, Italy; ^3^Centre of Excellence for Biomedical Research (CEBR), University of Genoa, Genoa, Italy; ^4^Immunology Operative Unit, IRCCS San Martino Polyclinical Hospital, Genoa, Italy; ^5^Department of Immunology, IRCCS Bambino Gesù Children’s Hospital, Rome, Italy; ^6^Department of Integrated Oncological Therapies, IRCCS San Martino Polyclinical Hospital, Genoa, Italy; ^7^Department of Earth Science, Environment and Life (DISTAV), University of Genoa, Genoa, Italy.

**Corresponding author**: Prof. Emanuela Marcenaro, Department of Experimental Medicine (DIMES) and Centre of Excellence for Biomedical Research (CEBR), University of Genoa, Via G.B. Marsano 10, 16132 Genoa, Italy. Phone: +39-010-3537888; Fax: +39-010-3537576; E-mail: [emanuela.marcenaro@unige.it](mailto:emanuela.marcenaro@unige.it)

**Co-corresponding author**: Prof. Simona Sivori, Department of Experimental Medicine (DIMES) and Centre of Excellence for Biomedical Research (CEBR), University of Genoa, Italy. Phone: +39-010-3537888; Fax: +39-010-3537576; E-mail: [simona.sivori@unige.it](mailto:emanuela.marcenaro@unige.it)

**Supplementary File 1**

**Normalized log2 miRNAs data before and after batch correction showing regulated miRNAs with the standard selection criteria**

Multi-table file containing the results of a bioinformatics analysis of a microarray project from Miltenyi Biotech performed on twenty human cell samples. The different sheets show: (**raw log2 data**) the original miRNA intensity values, (**normalized log2 data, normalized log2 data bc**) normalized log2 intensity values before and after batch correction respectively, (**expressed 1LU**) the dataset limited to miRNAs that showed an intensity of detection of at least 1LU in 5 donors or more in either CD56^bright^ or CD56^dim^ NK cells, and (**all regulated**) a selection of miRNAs filtered for a difference in the median expression level of at least two-fold in the CD56^bright^ group relative to the CD56^dim^ group and an adjusted p-value ≤ 0.05 obtained as a result of a t-test matching each CD56^bright^ sample with his own CD56^dim^ sample derived from the same donor. MiRNA expression was analyzed on Agilent Human microRNA Microarrays 8x60K v19.
