# Supplementary material for: New miRNA Signature Heralds Human NK Cell Subsets at Different Maturation Steps: Involvement of miR-146a-5p in the Regulation of KIR Expression
Source: Front Immunol. 2018 Oct 15;9:2360. doi: 10.3389/fimmu.2018.02360 (PMC6196268; doi:10.3389/fimmu.2018.02360)
Supplement: Supplementary file 3 [file Data_Sheet_3.ZIP › Supplementary File 3/Legend Supplementary File 3.docx]

**New miRNA signature heralds human NK cell subsets at different maturation steps: involvement of miR-146a-5p in the regulation of KIR expression**

**^1^Silvia Pesce^#^, ^2^Margherita Squillario^#^, ^1,3^Marco Greppi^#^, ^4^Fabrizio Loiacono, ^5^Lorenzo Moretta, ^1,3^Alessandro Moretta**^†^**, ^1,3^Simona Sivori, ^6^Patrizio Castagnola, ^2^Annalisa Barla*****, ^7^Simona Candiani*****, ^1,3^Emanuela Marcenaro***

^#^Equally contributed to this study

*These authors share senior authorship

^†^We dedicate this contribution to Alessandro Moretta who sadly passed away in mid February. We mourn his invaluable scientific insight and mentorship and, even more, his humanity, irony and smile.

^1^Department of Experimental Medicine (DIMES), University of Genoa, Genoa, Italy; ^2^Department of Informatic Bioengeneering, Robotic and System Engeneering, University of Genoa, Genoa, Italy; ^3^Centre of Excellence for Biomedical Research (CEBR), University of Genoa, Genoa, Italy; ^4^Immunology Operative Unit, IRCCS San Martino Polyclinical Hospital, Genoa, Italy; ^5^Department of Immunology, IRCCS Bambino Gesù Children’s Hospital, Rome, Italy; ^6^Department of Integrated Oncological Therapies, IRCCS San Martino Polyclinical Hospital, Genoa, Italy; ^7^Department of Earth Science, Environment and Life (DISTAV), University of Genoa, Genoa, Italy.

**Corresponding author**: Prof. Emanuela Marcenaro, Department of Experimental Medicine (DIMES) and Centre of Excellence for Biomedical Research (CEBR), University of Genoa, Via G.B. Marsano 10, 16132 Genoa, Italy. Phone: +39-010-3537888; Fax: +39-010-3537576; E-mail: [emanuela.marcenaro@unige.it](mailto:emanuela.marcenaro@unige.it)

**Co-corresponding author**: Prof. Simona Sivori, Department of Experimental Medicine (DIMES) and Centre of Excellence for Biomedical Research (CEBR), University of Genoa, Italy. Phone: +39-010-3537888; Fax: +39-010-3537576; E-mail: [simona.sivori@unige.it](mailto:emanuela.marcenaro@unige.it)

**Supplementary File 3**

**Common and specific KEGG Pathways**

Multi-table file containing a complete list of KEGG Pathways that results enriched in genes contained in Supplementary File 2 with the Webtool WebGestalt 2013 using “hsapiens_genome” as a reference set, Hypergeometric as a Statistical Method, Bonferroni Multiple Test Adjustment and showing results with a Significance Level ≤ 0.05 and a Minimum Numbers of Genes of 3. This test was run using the complete list of putative targets comprehensive of genes predicted from miRNAs regulated on CD56^bright^ and CD56^dim^ and was cross referenced with the results obtained using only the list of genes predicted from miRNAs regulated on either CD56^bright^ or CD56^dim^. The different sheets show: (**Common KEGG Pathways**) 195 Pathways in common between CD56^dim^ and CD56^bright^ putative target genes, (**KEGG Pathways CD56dim**) 2 Pathways specific for CD56^dim^ putative target genes and (**KEGG Pathways CD56bright**) 5 Pathways specific for CD56^bright^ putative target genes.
