# Supplementary material for: New miRNA Signature Heralds Human NK Cell Subsets at Different Maturation Steps: Involvement of miR-146a-5p in the Regulation of KIR Expression
Source: Front Immunol. 2018 Oct 15;9:2360. doi: 10.3389/fimmu.2018.02360 (PMC6196268; doi:10.3389/fimmu.2018.02360)
Supplement: Supplementary file 4 [file Presentation_1.PPTX]

## Slide 1
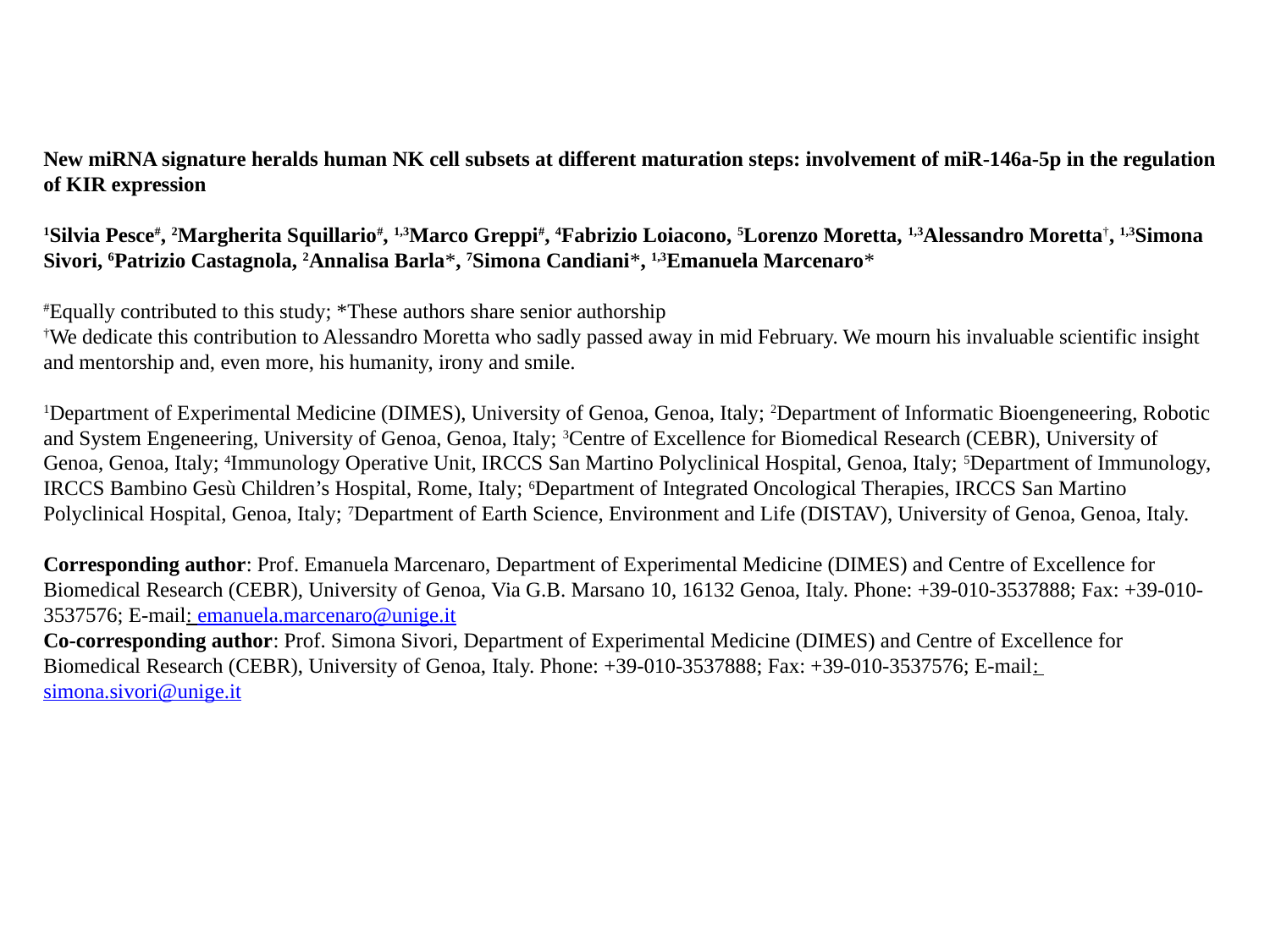

New miRNA signature heralds human NK cell subsets at different maturation steps: involvement of miR-146a-5p in the regulation of KIR expression
1Silvia Pesce#, 2Margherita Squillario#, 1,3Marco Greppi#, 4Fabrizio Loiacono, 5Lorenzo Moretta, 1,3Alessandro Moretta†, 1,3Simona Sivori, 6Patrizio Castagnola, 2Annalisa Barla*, 7Simona Candiani*, 1,3Emanuela Marcenaro*
#Equally contributed to this study; *These authors share senior authorship
†We dedicate this contribution to Alessandro Moretta who sadly passed away in mid February. We mourn his invaluable scientific insight and mentorship and, even more, his humanity, irony and smile.
1Department of Experimental Medicine (DIMES), University of Genoa, Genoa, Italy; 2Department of Informatic Bioengeneering, Robotic and System Engeneering, University of Genoa, Genoa, Italy; 3Centre of Excellence for Biomedical Research (CEBR), University of Genoa, Genoa, Italy; 4Immunology Operative Unit, IRCCS San Martino Polyclinical Hospital, Genoa, Italy; 5Department of Immunology, IRCCS Bambino Gesù Children’s Hospital, Rome, Italy; 6Department of Integrated Oncological Therapies, IRCCS San Martino Polyclinical Hospital, Genoa, Italy; 7Department of Earth Science, Environment and Life (DISTAV), University of Genoa, Genoa, Italy.
Corresponding author: Prof. Emanuela Marcenaro, Department of Experimental Medicine (DIMES) and Centre of Excellence for Biomedical Research (CEBR), University of Genoa, Via G.B. Marsano 10, 16132 Genoa, Italy. Phone: +39-010-3537888; Fax: +39-010-3537576; E-mail: emanuela.marcenaro@unige.it
Co-corresponding author: Prof. Simona Sivori, Department of Experimental Medicine (DIMES) and Centre of Excellence for Biomedical Research (CEBR), University of Genoa, Italy. Phone: +39-010-3537888; Fax: +39-010-3537576; E-mail: simona.sivori@unige.it

## Slide 2
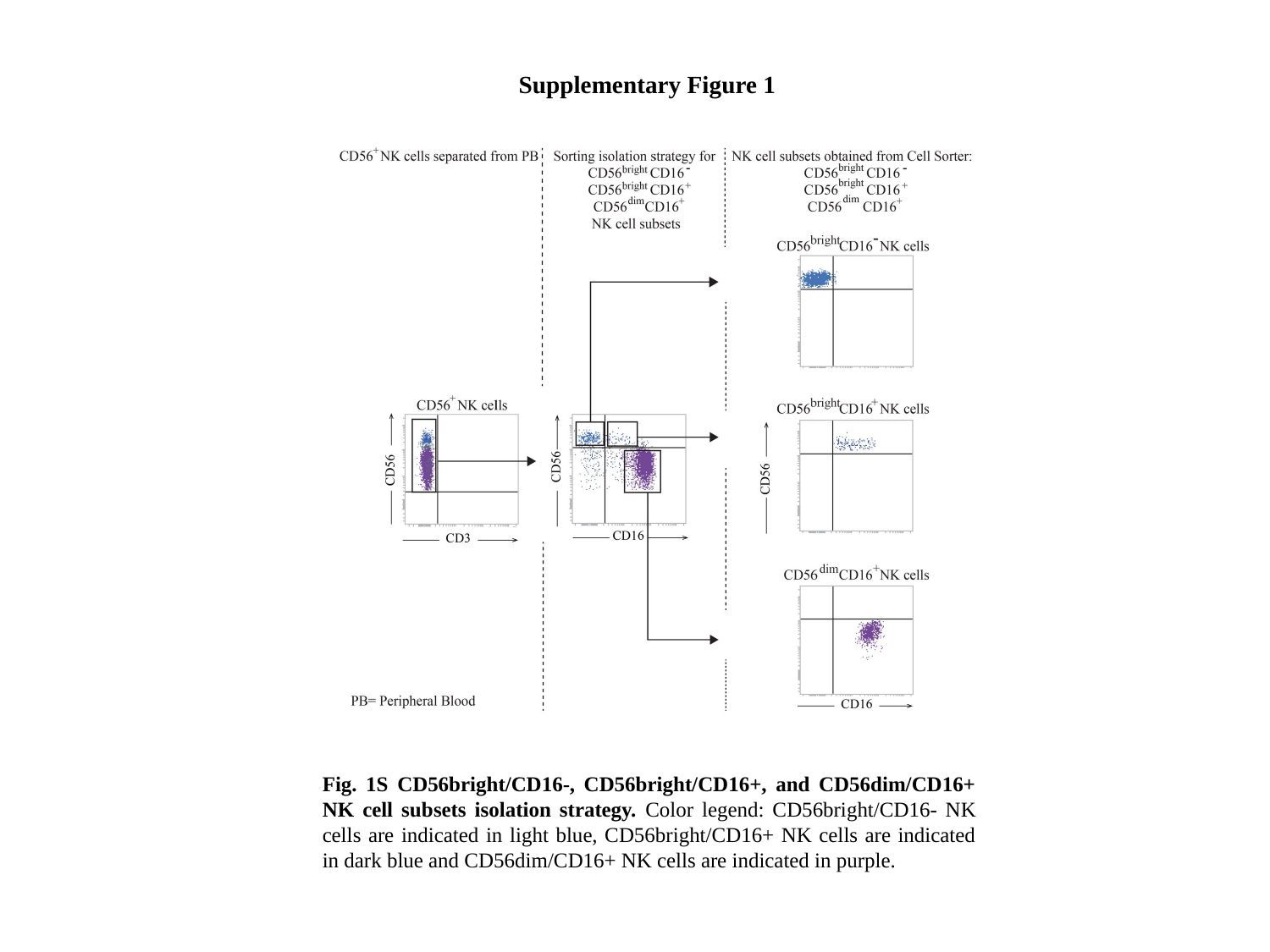

Supplementary Figure 1
Fig. 1S CD56bright/CD16-, CD56bright/CD16+, and CD56dim/CD16+ NK cell subsets isolation strategy. Color legend: CD56bright/CD16- NK cells are indicated in light blue, CD56bright/CD16+ NK cells are indicated in dark blue and CD56dim/CD16+ NK cells are indicated in purple.

## Slide 3
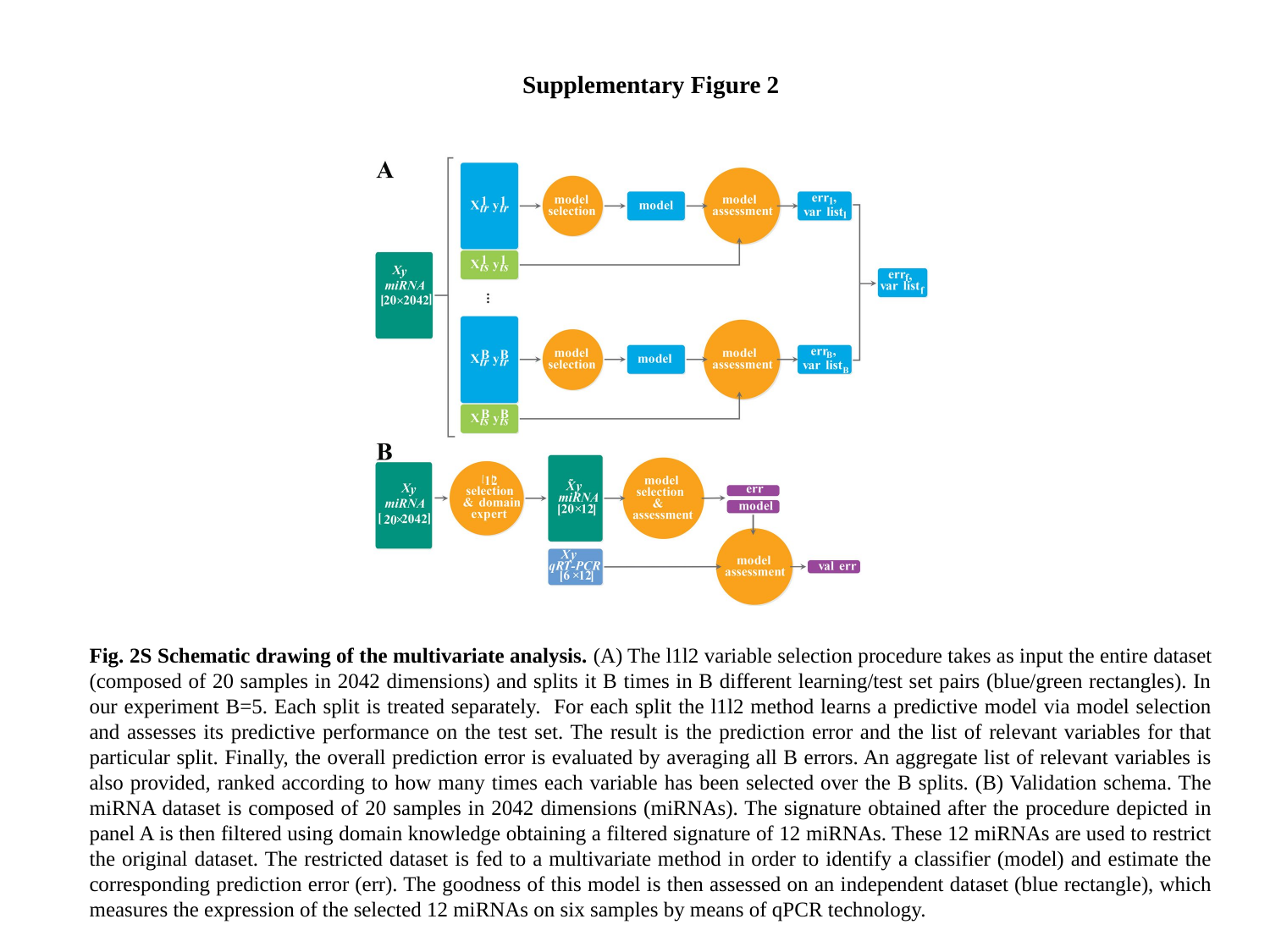

Supplementary Figure 2
Fig. 2S Schematic drawing of the multivariate analysis. (A) The l1l2 variable selection procedure takes as input the entire dataset (composed of 20 samples in 2042 dimensions) and splits it B times in B different learning/test set pairs (blue/green rectangles). In our experiment B=5. Each split is treated separately. For each split the l1l2 method learns a predictive model via model selection and assesses its predictive performance on the test set. The result is the prediction error and the list of relevant variables for that particular split. Finally, the overall prediction error is evaluated by averaging all B errors. An aggregate list of relevant variables is also provided, ranked according to how many times each variable has been selected over the B splits. (B) Validation schema. The miRNA dataset is composed of 20 samples in 2042 dimensions (miRNAs). The signature obtained after the procedure depicted in panel A is then filtered using domain knowledge obtaining a filtered signature of 12 miRNAs. These 12 miRNAs are used to restrict the original dataset. The restricted dataset is fed to a multivariate method in order to identify a classifier (model) and estimate the corresponding prediction error (err). The goodness of this model is then assessed on an independent dataset (blue rectangle), which measures the expression of the selected 12 miRNAs on six samples by means of qPCR technology.

## Slide 4
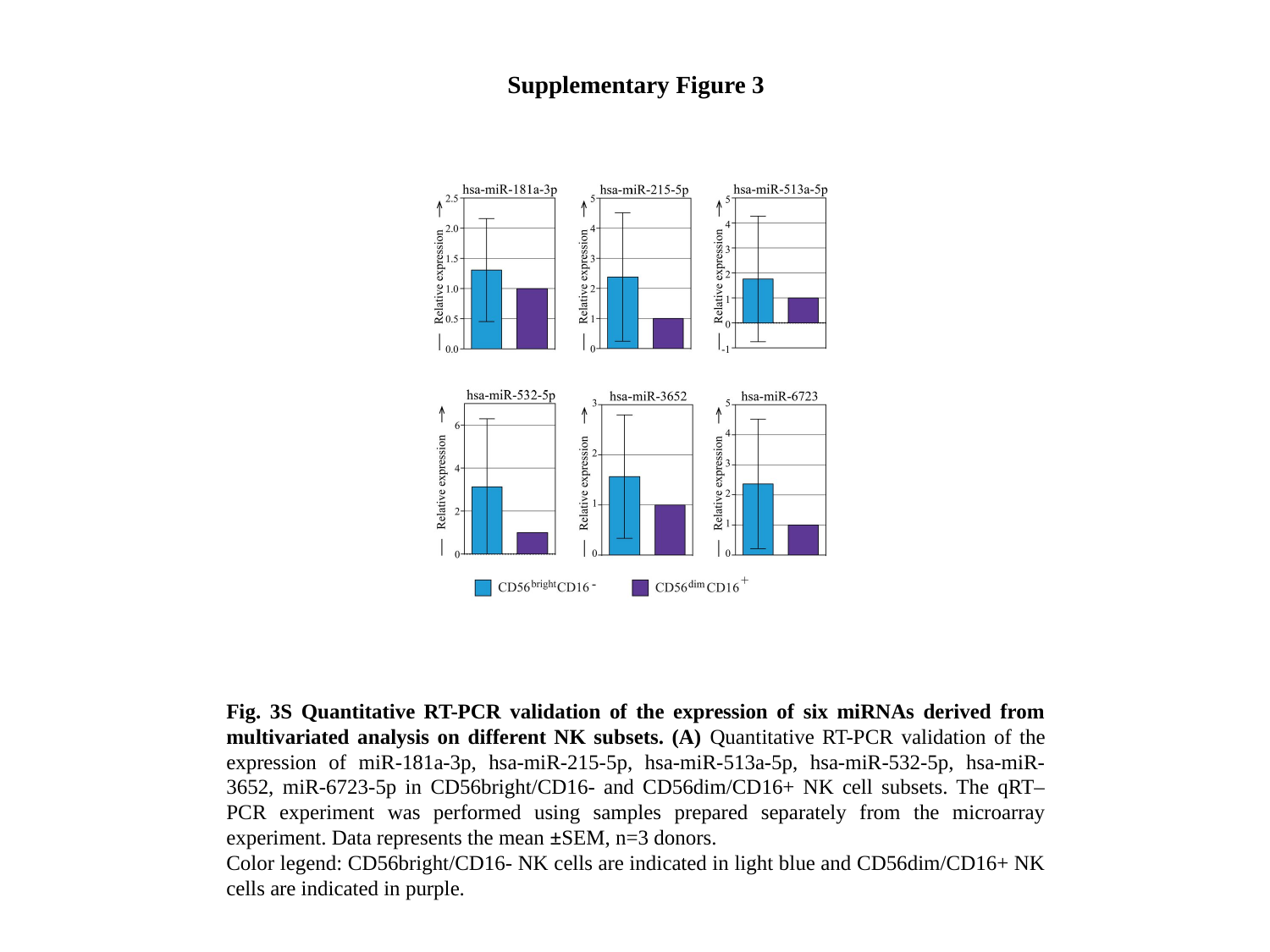

Supplementary Figure 3
Fig. 3S Quantitative RT-PCR validation of the expression of six miRNAs derived from multivariated analysis on different NK subsets. (A) Quantitative RT-PCR validation of the expression of miR-181a-3p, hsa-miR-215-5p, hsa-miR-513a-5p, hsa-miR-532-5p, hsa-miR-3652, miR-6723-5p in CD56bright/CD16- and CD56dim/CD16+ NK cell subsets. The qRT–PCR experiment was performed using samples prepared separately from the microarray experiment. Data represents the mean ±SEM, n=3 donors.
Color legend: CD56bright/CD16- NK cells are indicated in light blue and CD56dim/CD16+ NK cells are indicated in purple.
