# Supplementary material for: New miRNA Signature Heralds Human NK Cell Subsets at Different Maturation Steps: Involvement of miR-146a-5p in the Regulation of KIR Expression
Source: Front Immunol. 2018 Oct 15;9:2360. doi: 10.3389/fimmu.2018.02360 (PMC6196268; doi:10.3389/fimmu.2018.02360)
Supplement: Supplementary file 5 [file Table_1.DOCX]

**Table 1. miRNAs identified with multivariate analysis with different expression in CD56^bright^ and CD56^dim^.**

| **miRNAs identified by l1l2 which have a fold change < or > 2** | |
| --- | --- |
| **DIM** | **BRIGHT** |
| **hsa-miR-873-5p*** | **hsa-miR-31-5p*** |
| **hsa-miR-181a-2-3p*** | **hsa-miR-130a-3p*** |
| hsa-miR-152* | hsa-miR-133b* |
| hsa-miR-132-3p* | hsa-miR-31-3p* |
| hsa-miR-210* | hsa-miR-1* |
| hsa-miR-6723-5p | hsa-miR-218-5p* |
| hsa-miR-1271-5p* | hsa-miR-196b-5p* |
| hsa-miR-362-3p* | hsa-miR-99a-5p* |
| hsa-miR-373-5p | hsa-miR-151b* |
| hsa-miR-215 | hsa-miR-135b-5p* |
| hsa-miR-135a-3p | hsa-miR-125b-5p* |
| hsa-miR-1227-5p | hsa-miR-17-3p* |
| hsa-miR-718 | hsa-miR-151a-3p* |
| hsa-miR-33a-5p* | hsa-miR-20a-3p* |
| hsa-miR-200c-3p* | **hsa-miR-223-3p*** |
| hsa-miR-34a-5p | hsa-miR-652-3p* |
| hsa-miR-532-5p* | hsa-miR-5581-5p |
| hsa-miR-3652 | **hsa-miR-146a-5p*** |
| hsa-miR-181d | hsa-miR-151a-5p* |
| hsa-miR-4532 | hsa-miR-625-5p* |
| hsa-miR-876-5p* | hsa-miR-513a-5p |
| hsa-miR-4741 | hsa-miR-4716-3p |
| hsa-miR-4734 | hsa-miR-340-5p |
| hsa-miR-505-3p* | hsa-miR-181a-3p |
| hsa-miR-10a-5p | hsa-let-7e-5p |
| hsa-miR-29c-5p | hsa-miR-664b-5p |
| hsa-miR-32-5p | hsa-miR-1246 |
| hsa-miR-3937 | hsa-miR-642b-3p |
| hsa-miR-505-5p | hsa-miR-497-5p |
| hsa-miR-876-3p | hsa-miR-18a-5p* |
| hsa-miR-29b-1-5p | hsa-miR-195-5p* |
| has-miR-4634 | **hsa-miR-92a-3p*** |
| hsa-miR-7-5p* | hsa-miR-20a-5p* |
| hsa-miR-6722-3p |  |
| hsa-miR-4687-3p* |  |
| hsa-miR-1299 |  |
| hsa-miR-4788 |  |

**Table 1. miRNAs identified with multivariate analysis with different expression in CD56^bright^ and CD56^dim^.** List of miRNAs identified by l_1_l_2_ which have a fold change < or > 2 (37 miRNAs up-regulated on CD56^dim^ and 33 miRNAs up-regulated on CD56^bright^). Asterisks indicate those miRNAs identified by both univariate and multivariate analysis; 7 of the most regulated miRNAs are indicated in bold.
